# Single-cell analysis of pyroptosis dynamics reveals conserved GSDMD-mediated subcellular events that precede plasma membrane rupture

**DOI:** 10.1038/s41418-018-0106-7

**Published:** 2018-04-17

**Authors:** Nathalia M. de Vasconcelos, Nina Van Opdenbosch, Hanne Van Gorp, Eef Parthoens, Mohamed Lamkanfi

**Affiliations:** 1 Department of Internal Medicine, Ghent University, Ghent, B-9000 Belgium; 2 VIB-UGhent Center for Inflammation Research, VIB, Ghent, B-9000 Belgium; 3 VIB Bioimaging Core, VIB, Ghent, B-9000 Belgium; 4 Present Address: Janssen Immunosciences, World Without Disease Accelerator, Janssen Pharmaceutical Companies of Johnson and Johnson, Beerse, B-2340 Belgium

**Keywords:** Inflammasome, Immune cell death

## Abstract

Pyroptosis is rapidly emerging as a mechanism of anti-microbial host defense, and of extracellular release of the inflammasome-dependent cytokines interleukin (IL)-1β and IL-18, which contributes to autoinflammatory pathology. Caspases 1, 4, 5 and 11 trigger this regulated form of necrosis by cleaving the pyroptosis effector gasdermin D (GSDMD), causing its pore-forming amino-terminal domain to oligomerize and perforate the plasma membrane. However, the subcellular events that precede pyroptotic cell lysis are ill defined. In this study, we triggered primary macrophages to undergo pyroptosis from three inflammasome types and recorded their dynamics and morphology using high-resolution live-cell spinning disk confocal laser microscopy. Based on quantitative analysis of single-cell subcellular events, we propose a model of pyroptotic cell disintegration that is initiated by opening of GSDMD-dependent ion channels or pores that are more restrictive than recently proposed GSDMD pores, followed by osmotic cell swelling, commitment of mitochondria and other membrane-bound organelles prior to sudden rupture of the plasma membrane and full permeability to intracellular proteins. This study provides a dynamic framework for understanding cellular changes that occur during pyroptosis, and charts a chronological sequence of GSDMD-mediated subcellular events that define pyroptotic cell death at the single-cell level.

## Introduction

Pyroptosis is a lytic form of regulated cell death that is induced by inflammatory caspases 1, 4, 5 and 11 [1, 2]. Murine caspase-11 and its human orthologs caspases 4 and 5 are activated by cytosolic lipopolysaccharides (LPS), and indirectly promote activation of caspase-1 through the non-canonical inflammasome pathway [3]. Caspase-1 cleaves the biologically inert precursor proteins interleukin (IL)-1β and IL-18 into the mature, secreted inflammatory cytokines [4]. Unlike for IL-1β and IL-18, each of the aforementioned inflammatory caspases can induce pyroptosis directly by cleaving gasdermin D (GSDMD) at the central linker peptide, which separates the pore-forming amino-terminal domain (GSDMD_N_) from the inhibitory carboxy-terminal (GSDMD_C_) domain [5–8]. This cleavage event causes GSDMD_N_ to oligomerize and insert in the plasma membrane, giving rise to rapid cell lysis.

Pyroptosis as a cell biological phenomenon was first reported in the context of macrophages that had been infected with the Gram-negative bacterial pathogens *Shigella flexneri* and *Salmonella enterica* serovar Typhimurium (*S*. Typhimurium), respectively [9–11]. However, the term pyroptosis was coined only in 2001 to distinguish this inflammatory form of caspase-regulated necrosis from accidental necrosis and apoptosis [12]. To the best of our knowledge, there has been no detailed characterization at the single-cell level of the morphology and subcellular dynamics that precede pyroptotic cell lysis. Here, we triggered primary macrophages to undergo pyroptosis from three inflammasome types and recorded their dynamics and morphology using high-resolution live-cell spinning disk confocal laser microscopy. Our findings chart a chronological sequence of subcellular events that define pyroptotic cell death at the single-cell level.

## Results

### Myosin II-independent blebbing and differential cell detachment during pyroptosis and necroptosis

**Fig. 1:**
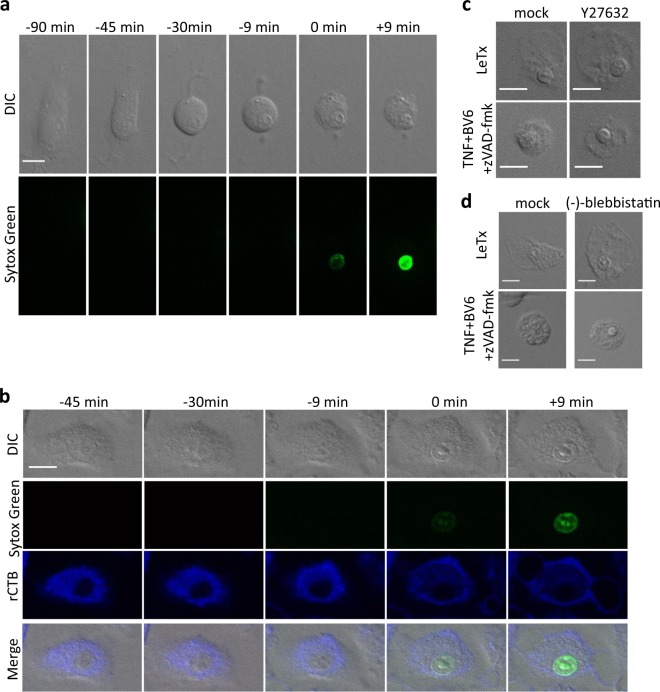
Cell detachment and blebbing during necroptosis and pyroptosis. **a** B6^Nlrp1b+^ BMDMs were stimulated with TNF+BV6+zVAD-fmk (TBz: 20 ng/ml, 2 µM and 50 µM, respectively) and imaged in culture media containing Sytox Green. **b** The rCTB-stained B6^Nlrp1b+^ BMDMs were stimulated with LeTx and imaged as in (**a**). **a**,** b** Confocal images were acquired every 3 min. **c**, **d** B6^Nlrp1b+^ BMDMs pretreated with Y27632 (10 μM) (**c**) or (−)-blebbistatin (10 μM) (**d**) were stimulated with LeTx or TBz and imaged. Fluorescent micrographs show the maximum intensity projection (Sytox Green) or the single plane (rCTB) of a representative cell from 3 independent experiments (TBz *n* = 30; LeTx *n* = 30; LeTx+Y27632 *n* = 25; TBz+Y27632 *n* = 23; LeTx+(−)-blebbistatin *n* = 19; TBz+(−)-blebbistatin *n* = 20). In all panels, time point zero indicates the first detection of Sytox Green. All scale bars, 10 µm

Pyroptosis and necroptosis are both lytic forms of regulated cell death, but it is unclear whether they differ in particular features. C57BL/6J mouse bone marrow-derived macrophages (BMDMs) that express a functional NLRP1b allele (B6^Nlrp1b+^) can be induced to undergo caspase-1-dependent pyroptosis when stimulated with *Bacillus anthracis* lethal toxin (LeTx) [13]. Stimulation with murine Tumor Necrosis Factor (TNF)+BV6+zVAD-fmk (TBz) induces necroptosis in macrophages and other cell types [14]. We used these cytotoxic stimuli to compare morphological features of B6^Nlrp1b+^ BMDMs undergoing necroptosis and pyroptosis. As previously reported in necroptotic L929sAhFas cells [15], TBz-treated B6^Nlrp1b+^ macrophages readily detached from the adherent surface and rounded up prior to losing plasma membrane integrity and becoming Sytox Green positive (Fig. 1a and Supplemental Movie 1). The membrane appeared smooth during this process, and formation of balloon-like protrusions of the plasma membrane that were reminiscent of blebs were seen concomitant with the loss of plasma membrane integrity (Fig. 1a). Unlike necroptotic cells, pyroptotic macrophages remained attached to the adherent surface until they became Sytox Green positive (Fig. 1b and Supplemental Movie 2). As during necroptosis, however, plasma membrane rupture was accompanied by the formation of blebs (Fig. 1b). The ROCK-I inhibitor Y27632 and the selective inhibitor of non-muscle myosin II ATPases (−)-blebbistatin inhibited blebbing in apoptotic cells (data not shown). However, inhibition of ROCK-I and myosin-II had no effect on pyroptotic and necroptotic blebbing (Fig. 1c, d).

### Phosphatidylserine exposure is closely associated with plasma membrane rupture during pyroptosis

**Fig. 2:**
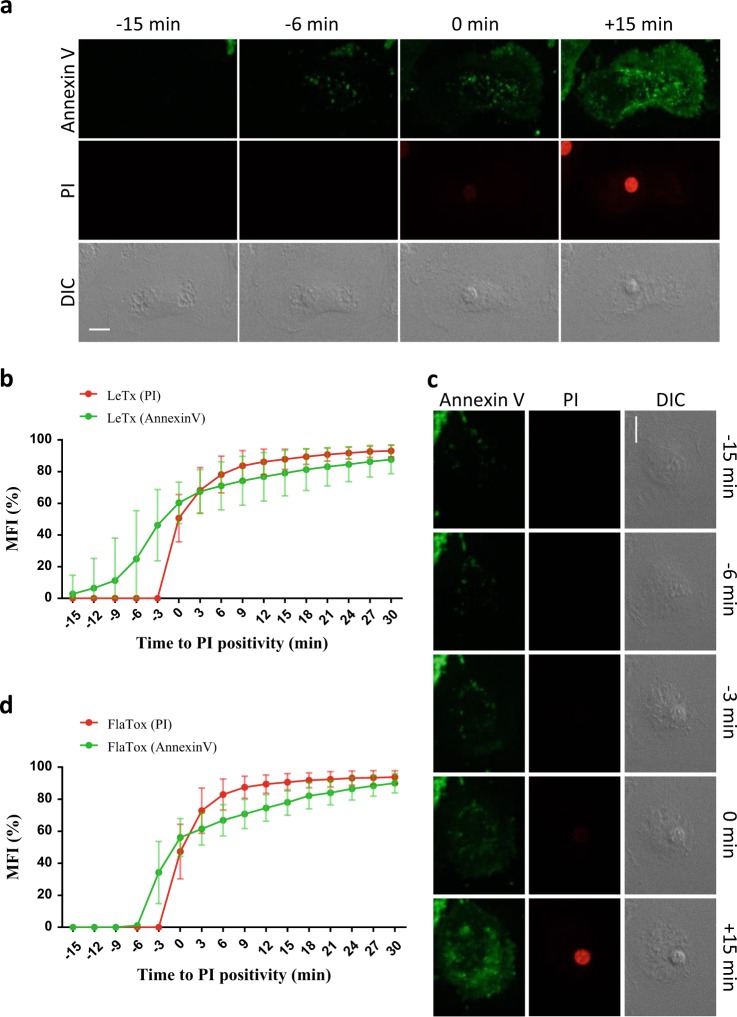
PS exposure happens during pyroptosis. **a**–**d** B6^Nlrp1b+^ BMDMs were stimulated with LeTx (**a**, **b**) or FlaTox (**c**,** d**) and imaged in culture media containing Annexin-V–FITC and PI. Confocal images were acquired every 3 min. Graphs show the percentage of mean fluorescence intensity (MFI) calculated as described in the Methods section, and values represent the mean ± SD of all individual cells that were imaged in three independent experiments (LeTx *n* = 18; FlaTox *n* = 21). Single-cell plots are shown in Supplemental Fig. 1. Fluorescent micrographs show the maximum intensity projection of a representative. In all panels, time point zero indicates the first detection of PI. All scale bars, 10 µm

Early phosphatidylserine exposure is a hallmark of apoptosis that attracts engulfing cells [16]. Recent studies showed that also mammalian cell lines undergoing necroptosis, as well as necrotic cells in the nematode *Caenorhabditis elegans* actively present phosphatidylserine on their outer surfaces prior to cell lysis to recruit phagocytes [17, 18]. To study the kinetics of phosphatidylserine exposure relative to plasma membrane rupture during pyroptosis, LeTx-treated B6^Nlrp1b+^ BMDMs were quantified for Annexin-V–fluorescein isothiocyanate (FITC) and propidium iodide (PI) positivity over time. This analysis indicated that Annexin-V positivity preceded plasma membrane rupture in approximately 9–12 min during LeTx-induced pyroptosis (Fig. 2a, b, Supplemental Fig. 1a and Supplemental Movie 3). Exposure of untreated B6^Nlrp1b+^ BMDMs failed to yield signals for Annexin-V and PI during this time interval, demonstrating specificity of these findings (Supplemental Movie 4).

To understand whether Annexin-V staining prior to cell lysis is a shared feature of pyroptosis when routed through other inflammasomes, we repeated the analysis for LFn-FlaA-treated B6^Nlrp1b+^ BMDMs. LFn-FlaA selectively triggers NAIP5/NLRC4 inflammasome-dependent pyroptosis and consists of *Legionella pneumophila* flagellin (FlaA) fused to the N-terminal domain of *B. anthracis* lethal factor (LFn) to enable *B. anthracis* protective antigen (PA)-assisted cytosolic delivery of the fusion protein [19]. Quantification of data from 21 cells from several independent experiments showed that Annexin-V staining was observed in B6^Nlrp1b+^ BMDMs treated with PA+LFn-FlaA (here called FlaTox) approximately 3 min before cells became PI positive (Fig. 2c, d, Supplemental Fig. 1b and Supplemental Movie 5). Future studies will need to address whether this corresponds to active phosphatidylserine exposure to the outer leaflet of the plasma membrane as our confocal micrographs did not allow differentiation between inner and outer staining of phosphatidylserine. We conclude from these studies that phosphatidylserine exposure occurs in pyroptosis in close association with plasma membrane rupture.

### Mitochondrial commitment independently of the apoptotic Bax/Bak pore

**Fig. 3:**
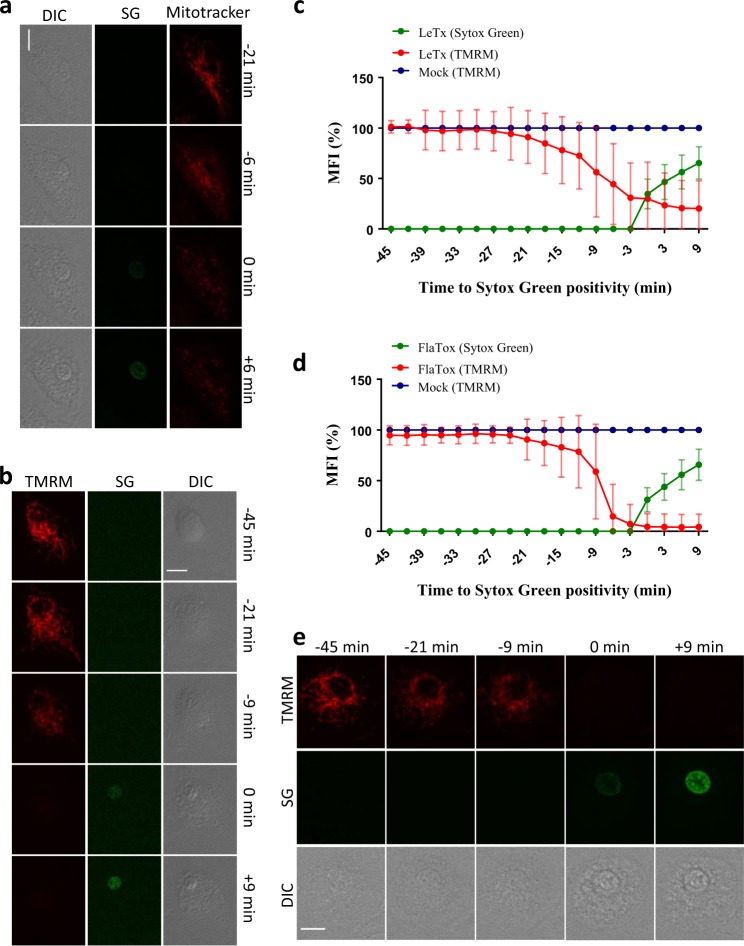
Mitochondria are damaged during pyroptosis. **a** B6^Nlrp1b+^ BMDMs were preloaded with Mitotracker Red CMXRos and stimulated with LeTx in culture media containing Sytox Green (*n* = 50). Confocal images were acquired every 3 min. **b**–**e** B6^Nlrp1b+^ BMDMs were preloaded with TMRM and stimulated with either LeTx (**b**,** c**) or FlaTox (**d**,** e**) and imaged as in (**a**). Graphs show the percentage of mean fluorescence intensity (MFI) calculated as described in the Methods section, and values represent the mean ± SD of all individual cells that were imaged in five independent experiments (LeTx, *n* = 28; FlaTox *n* = 28). Single-cell plots are shown in Supplemental Fig. 3. Fluorescent micrographs show the maximum intensity projection (TMRM and Sytox Green) or the single plane (Mitotracker) of a representative cell. In all panels, time point zero indicates the first detection of Sytox Green. All scale bars, 10 µm

Mitochondria play a central role during apoptosis [20]. In contrast, the role of mitochondria in inflammasome signaling is contentious with mitochondrial damage being proposed to occur upstream or downstream of the NLRP3 and AIM2 inflammasomes [21, 22]. We performed a longitudinal live-cell analysis of mitochondrial dynamics to better understand the role of this organelle in pyroptosis. Active mitochondria of B6^Nlrp1b+^ macrophages were labeled with the membrane potential (ΔΨ_m_)-insensitive dye MitoTracker Red CMXRos. Consistent with mitochondrial commitment during pyroptosis, the tubular mitochondrial network seen in mock-treated cells (Supplemental Fig. 2) was lost following LeTx challenge (Fig. 3a and Supplemental Movie 6). Notably, mitochondria appeared rounded and fragmented before cell rupture, as marked by Sytox Green internalization (Fig. 3a). A quantitative analysis of tetramethylrhodamine (TMRM) fluorescence, a mitochondrial membrane potential (ΔΨ_m_)-sensitive dye, confirmed that LeTx-induced mitochondrial membrane depolarization occurred approximately 18–21 min before plasma membrane rupture (Fig. 3b, c, Supplemental Fig. 3a and Supplemental Movie 7). As reported for the AIM2 and NLRP3 inflammasomes [23], the pro-apoptotic Bcl2 family member BID was cleaved when pyroptosis was induced in LeTx-intoxicated B6^Nlrp1b+^ macrophages (Supplemental Fig. 4a). However, LeTx-induced caspase-1 maturation and lactate dehydrogenase (LDH) release were unaffected in B6^Nlrp1b+^ BMDMs from transgenic mice that overexpress the Bax/Bak pore antagonist Bcl2 under control of the H2K promoter (Supplemental Fig. 4b, c). Bcl2-transgenic BMDMs were also shown to induce normal caspase-1 activation and IL-1β secretion in response to NLRP3-activating stimuli [21]. Moreover, we found that the kinetics of LeTx-induced TMRM signal decay in Bcl2-overexpressing B6^Nlrp1b+^ macrophages was comparable to that of B6^Nlrp1b+^ macrophages, consistent with mitochondrial outer membrane permeabilization during pyroptosis occurring independently of the Bax/Bak pore (Supplemental Fig. 4d, e). Notably, the kinetics of mitochondrial decay induced by the NLRC4 inflammasome was remarkably consistent with that of LeTx-treated macrophages, both featuring a loss of TMRM signal on average 20 min before plasma membrane rupture (Fig. 3d, e, Supplemental Fig. 3b and Supplemental Movie 8). Thus, Bcl2-insensitive mitochondrial damage is a conserved feature of pyroptotic cell dismantling.

### Lysosome decay precedes pyroptotic plasma membrane rupture

**Fig. 4:**
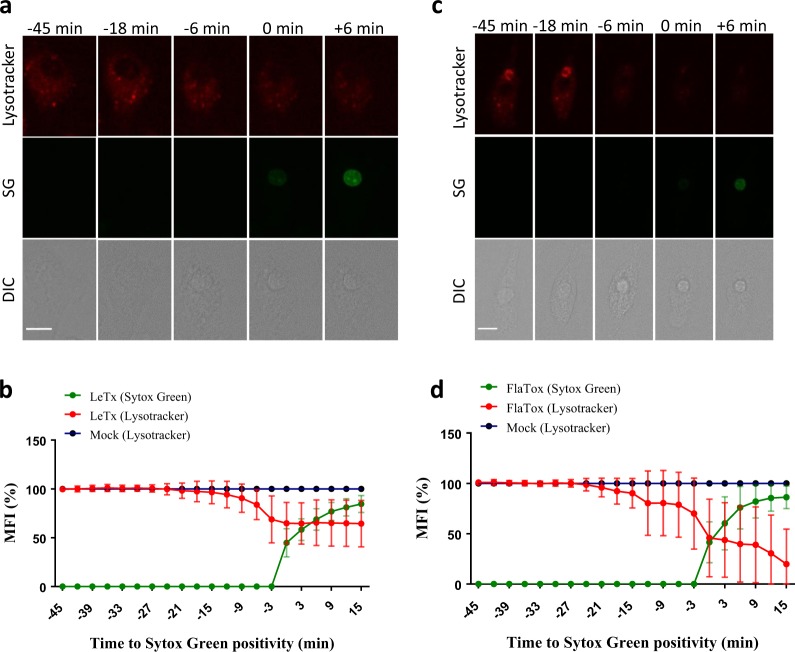
Lysosomes decay prior to pyroptotic cell lysis. **a**–**d** B6^Nlrp1b+^or B6 BMDMs preloaded with Lysotracker and stimulated with LeTx (**a**,** b**) or FlaTox (**c**,** d**), respectively, were imaged throughout cell death in culture media containing Sytox Green. Confocal images were taken every 3 min. Graphs show the percentage of mean fluorescence intensity (MFI) calculated as described in the Methods section, and values represent the mean ± SD of all individual cells that were imaged in 3 independent experiments (LeTx, *n* = 27; FlaTox *n* = 19). Single-cell plots are shown in Supplemental Fig. 5. Fluorescent micrographs show the maximum intensity projection of a representative cell out of at least 19 imaged cells. In all panels, time point zero indicates the first detection of Sytox Green. All scale bars, 10 µm

To document the fate of lysosomes during pyroptosis, we stained B6^Nlrp1b+^ macrophages with LysoTracker, a fluorescent probe that is highly selective for acidic organelles, and imaged the lysosomal fluorescence over time. Lysosome staining remained relatively stable following LeTx stimulation until LysoTracker fluorescence declined gradually 6–9 min before the plasma membrane ruptured (Fig. 4a, b, Supplemental Fig. 5a and Supplemental Movie 9). These results are in line with a previous report demonstrating that LeTx induced a loss of lysosomal acidity which depended on expression of a LeTx-responsive NLRP1b allele [24]. Lysosome decay was not restricted to LeTx because we also observed a decrease in LysoTracker staining when pyroptosis was induced through the NLRC4 inflammasome (Fig. 4c, d, Supplemental Fig. 5b and Supplemental Movie 10). The kinetics of lysosome destabilization in FlaTox-treated C57BL/6J (B6) macrophages was closely aligned with that of LeTx-intoxicated cells, with LysoTracker signal decay occurring around 6–9 min before cells became Sytox Green-positive (Fig. 4c, d). Together, these results demonstrate that lysosome decay is a conserved feature of pyroptosis that precedes plasma membrane damage by about 10 min.

### Nuclear rounding and condensation are late-stage pyroptotic events

**Fig. 5:**
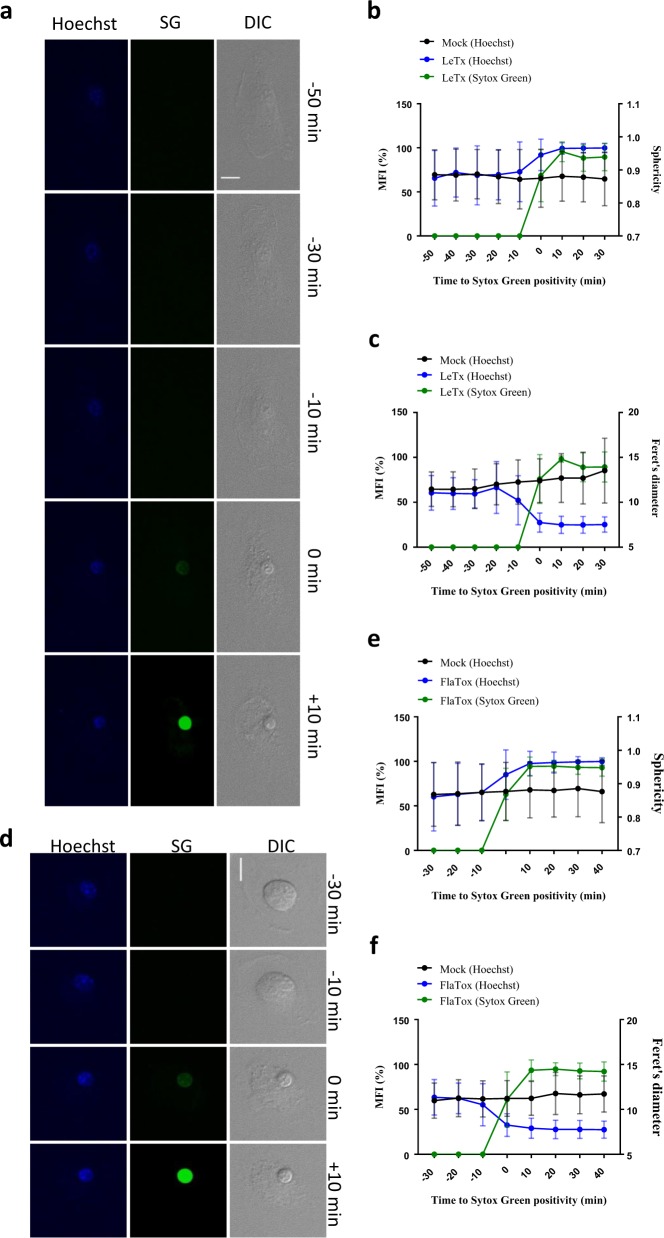
Nuclei round up and condense during pyroptosis. **a**–**e** B6^Nlrp1b+^ BMDMs were preloaded with Hoechst dye and stimulated with LeTx (**a**–**c**) or FlaTox (**c**–**e**) before imaging in culture media containing Sytox Green. Confocal images were acquired every 10 min. Graphs show the percentage of mean fluorescence intensity (MFI) of Sytox Green and nuclear sphericity or the Feret’s diameter based on Hoechst staining, all calculated as described in the Methods section. Values represent the mean ± SD of individual cells imaged in three independent experiments (LeTx: Sphericity *n* = 24, Feret’s diameter *n* = 18; FlaTox: Sphericity *n* = 26, Feret’s diameter *n* = 20). Single-cell plots are shown in Supplemental Fig. 6. Fluorescent micrographs show the maximum intensity projection of a representative cell. In all panels, time point zero indicates the first detection of Sytox Green. All scale bars, 10 µm

Nuclear condensation and internucleosomal DNA fragmentation are hallmark features of apoptosis. Early studies of pyroptosis suggested that caspase-1 activation in *S*. Typhimurium-infected macrophages triggers a diffuse pattern of DNA fragmentation in the absence of nuclear condensation [9, 25]. In agreement, several reports documented terminal deoxynucleotidyl transferase-mediated dUTP-biotin nick end-label (TUNEL) activity in *S*. Typhimurium-infected macrophages [9, 25–27]. However, little is known about the dynamics of early- and late-stage nuclear events during pyroptosis. We stained nuclear DNA of B6^Nlrp1b+^ macrophages with Hoechst 33342 (Hoechst) stain, and imaged cells to track the shape and size of the nucleus following LeTx challenge. Pyroptotic macrophages retained an ellipsoid-shaped nucleus until they became Sytox Green positive (Fig. 5a and Supplemental Movie 11). In agreement, examination of sphericity—a measure of how close the shape of an object is to a perfect sphere—using the Imaris microscopy image analysis platform confirmed that the macrophage nucleus rounded up concurrently to the loss of plasma membrane integrity (Fig. 5b and Supplemental Fig. 6a). From these analyses, we also noted that the nucleus appeared more condensed following plasma membrane rupture. Consistently, a kinetic analysis of the nucleus’ Feret diameter—a measure of an object size along a specified direction—showed that nuclear condensation is a late-stage pyroptotic event that occurs together with plasma membrane rupture (Fig. 5c and Supplemental Fig. 6b). Similarly, pyroptosis induction in FlaTox-treated B6^Nlrp1b+^ macrophages was accompanied by nuclear rounding and condensation around the time when cells turned Sytox Green positive (Fig. 5d–f, Supplemental Fig. 6c, d and Supplemental Movie 12). These results demonstrate that nuclear rounding and condensation are late-stage pyroptotic events.

### Cell swelling during pyroptosis

Cell volume depends on the osmotic movement of water across the plasma membrane, which is fundamentally regulated by ion and voltage gradients and balanced by active ion transport across membranes [28]. Pyroptotic cells have long been recognized to swell [26], but the current model that pyroptosis is induced by the insertion in the plasma membrane of large pre-assembled non-selective GSDMD pores suggests that simultaneous exchange of ions and proteins across the plasma membrane should maintain osmolarity during pyroptosis [29]. To gain more insight into this process, we studied the cell volume dynamics of primary macrophages undergoing pyroptotic cell death.

**Fig. 6:**
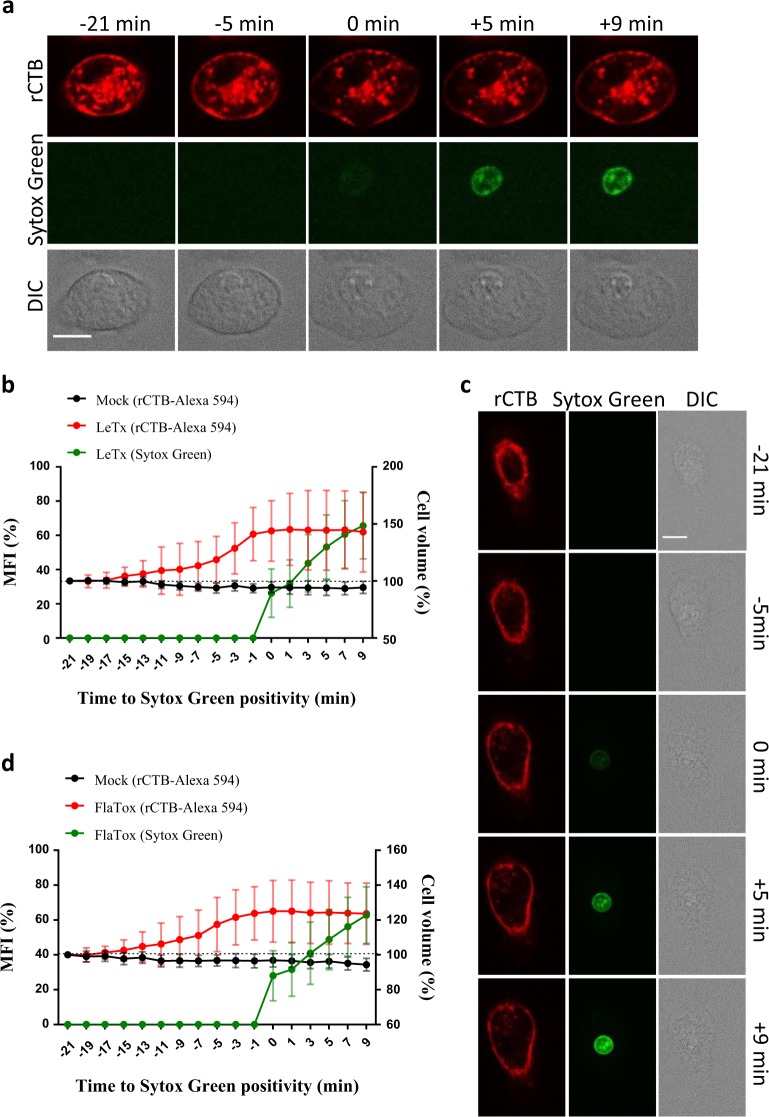
Cell swelling precedes pyroptotic cell rupture. **a**–**d** B6^Nlrp1b+^ or B6 BMDMs stained with Cholera Toxin subunit B-Alexa 594 (rCTB) were stimulated with LeTx (**a**,** b**) or FlaTox (**c**,** d**), respectively, and imaged in culture media containing Sytox Green. Confocal images were acquired every 1.5 min. Graphs show the percentage of mean fluorescence intensity (MFI) of Sytox Green and cell volume quantifications based on rCTB-Alexa 594 staining, both calculated as described in the Methods section. Values represent the mean ± SD of individual cells imaged in 3 independent experiments (LeTx, *n* = 26; FlaTox *n* = 16). Fluorescent micrographs show the maximum intensity projection (Sytox Green) or the single plane (rCTB) of a representative cell. Single-cell plots are shown in Supplemental Fig. 7. In all panels, time point zero indicates the first detection of Sytox Green. All scale bars, 10 µm

The plasma membrane of B6^Nlrp1b+^ macrophages was stained with Cholera Toxin Subunit B (rCTB) coupled to the fluorescent dye Alexa 594. Cells were subsequently stimulated with LeTx to induce pyroptosis by the NLRP1b inflammasome, and we recorded single-cell volume changes in real time until cell lysis was evident by Sytox Green staining. Contrary to mock-treated macrophages, LeTx induced a gradual increase in cell volume starting approximately 13 min before cell rupture (Fig. 6a, b, Supplemental Fig. 7a and Supplemental Movie 13). The cell volume increased by up to 50% before contracting again after cell lysis (Fig. 6a, b). Pyroptosis following FlaTox-induced activation of the NLRC4 inflammasome was also associated with cell swelling before cellular internalization of Sytox Green was observed (Fig. 6c, d, Supplemental Fig. 7b and Supplemental Movie 14). Under these conditions, cells swelled up to 30% (Fig. 6d). A recent study proposed the pomegranate-derived polyphenolic compound punicalagin to inhibit NLRP3 and AIM2 inflammasome-induced IL-1β secretion by preventing plasma membrane permeability downstream of inflammasome activation [30]. Punicalagin inhibited release of LDH in the culture medium and PI internalization by LeTx-intoxicated B6^Nlrp1b+^ macrophages (Supplemental Fig. 8a, f), but in our hands was associated with upstream blockade of caspase-1 maturation, calcium influx and cell swelling (Supplemental Fig. 8). Punicalagin also interfered with FlaTox-induced caspase-1 maturation and LDH release in a dose-dependent manner (Supplemental Fig. 9a-e), suggesting that this compound interferes with pyroptosis and IL-1β secretion by upstream blockage of inflammasome activation. Although these findings vitiated punicalagin’s further use for probing pyroptosis execution mechanisms, our results demonstrate that osmotic swelling occurs in advance of cell lysis.

### Pyroptosis features ion fluxing prior to cell lysis

**Fig. 7:**
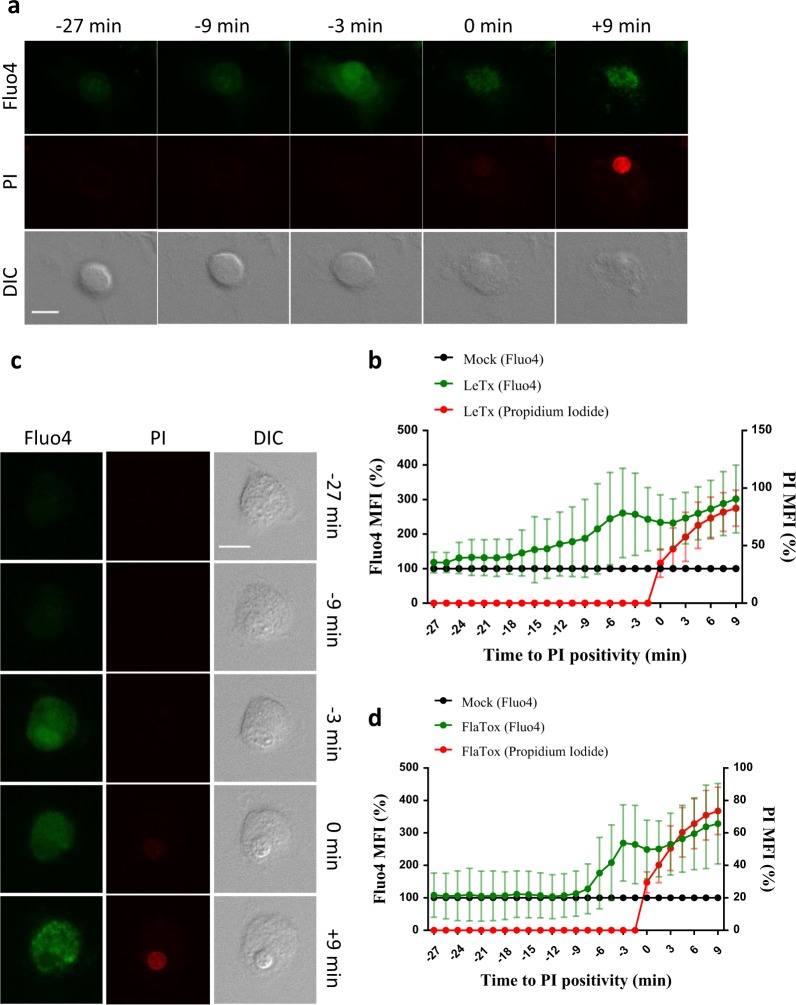
Ca^2+^ influx occurs prior to total membrane permeabilization in pyroptosis. **a**–**d** B6^Nlrp1b+^ BMDMs preloaded with the cell-permeant Ca^2+^ indicator Fluo4 were imaged after stimulation with LeTx (**a**,** b**) or FlaTox (**c**,** d**) in culture media containing PI. Confocal images were acquired every 1.5 min. Graphs show the percentage of mean fluorescence intensity (MFI) calculated as described in the Methods section, and values represent the mean ± SD of individual cells imaged in 4 independent experiments (LeTx, *n* = 24; FlaTox *n* = 23). Single-cell plots are shown in Supplemental Figure 10. Fluorescent micrographs show the maximum intensity projection of a representative cell. In all panels, time point zero indicates the first detection of PI. All scale bars, 10 µm

An early report estimated pyroptotic membrane pores in *S*. Typhimurium-infected macrophages to be 1.1–2.4 nm in diameter based on the size range of osmoprotectant molecules that prevented cell lysis [26]. More recent studies showed that GSDMD_N_ pores with inner diameters of 10–20 nm formed in liposomes, suggesting that pyroptosis may be associated with assembly of pyroptotic pores that are sufficiently large to theoretically allow passage of IL-1β, IL-18 and other cytosolic proteins [6, 7, 31, 32]. However, our observation that pyroptotic cells undergo cell volume increase up to 10 min before total lysis (Fig. 6) suggested that pyroptotic cells have permeability of their plasma membrane prior to total lysis, as marked by Sytox Green. To probe the size dynamics of pyroptotic pores, LeTx-stimulated B6^Nlrp1b+^ macrophages were preloaded with Fluo4, a cell-permeant fluorogenic probe that fluoresces strongly upon Ca^2+^ binding, and tracked for internalization of Ca^2+^ (*M*_w_ = 40 Da; van der Waals radius = 0.23 nm) relative to uptake of the DNA-intercalating agent PI (*M*_w _= 668 Da). Notably, we observed an increase in Fluo4 staining that preceded PI incorporation by approximately 12–15 min (Fig. 7a, b, Supplemental Fig. 10a and Supplemental Movie 15). Echoing these results, induction of pyroptosis through the NLRC4 inflammasome in FlaTox-treated B6^Nlrp1b+^ BMDMs was also associated with Ca^2+^ entry prior to PI positivity. Under these conditions, an increased Fluo4 signal was noted 6–9 min before cells became PI positive (Fig. 7c, d, Supplemental Fig. 10b and Supplemental Movie 16), in line with FlaTox-induced cell volume increase being slightly delayed relative to LeTx-treated cells (Fig. 6). Interestingly, internalization of the DNA-intercalating agent ethidium bromide (*M*_w_ = 394 Da) occurred slightly ahead of Sytox Green (*M*_w_ = 609 Da) uptake (Supplemental Fig. 11 and Supplemental Movie 17), suggesting that pyroptotic cells sequentially provide passage to small molecules according to their molecular weight.

### GSDMD mediates early ionic flux and mitochondrial decay in pyroptotic cells

GSDMD_N_ pores were recently reported to have inner diameters of 10–20 nm when assembled in vitro in liposomes [6, 7, 31, 32]. However, our observations demonstrate that pyroptotic Ca^2+^ influx precedes plasma membrane rupture and involves a pore or channel that excludes entry of DNA-intercalating agents of *M*_w_ 400–600 Da. We hypothesized that early ionic flux and cell swelling during pyroptosis is mediated by GSDMD pores of lower stoichiometry while the larger structures that form in liposomes may represent a terminal steady-state phase of GSDMD pore assembly associated with catastrophic cell lysis. However, LeTx and FlaTox treatment of *GSDMD*^*-/-*^ macrophages induced apoptosis with kinetics approaching those of pyroptosis induction in GSDMD-sufficient B6^Nlrp1b+^ and B6 macrophages (Supplemental Fig. 12a, b), thus impeding examination of the role of GSDMD pores in early pyroptotic ion fluxing with the above canonical inflammasome agonists.

**Fig. 8:**
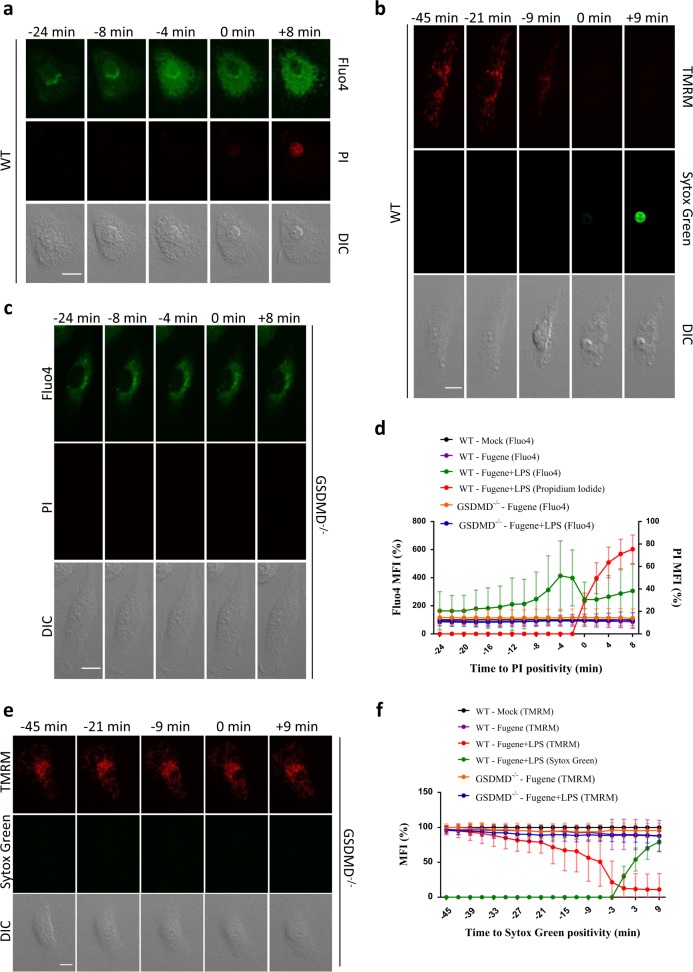
GSDMD deficiency prevents LPS transfection-induced early Ca^2+^ influx and mitochondrial decay. **a**,** c** Pam3csk4-primed BMDMs of the indicated genotypes were preloaded with the cell-permeant Ca^2+^ indicator Fluo4 and imaged after transfection with LPS (2 µg/ml, Fugene+LPS), treated with Fugene alone or 'mock'-treated in culture media containing PI. Confocal images were acquired every 2 min. Fluorescent micrographs show the maximum intensity projection of a representative cell. **b**,** e** BMDMs of the indicated genotypes were preloaded with TMRM and imaged after transfection with LPS (2 µg/ml, Fugene+LPS), treated with Fugene alone, or 'mock'-treated in culture media containing Sytox Green. Confocal images were acquired every 3 min. Fluorescent micrographs show the maximum intensity projection of a representative cell. **d**,** f** Graphs show the percentage of mean fluorescence intensity (MFI) calculated as described in the Methods section, and values represent the mean ± SD of individual cells imaged in 3 or 4 independent experiments (Fluo4: WT *n* = 18, GSDMD^-/-^ *n* = 28; TMRM: WT *n* = 18, GSDMD^-/-^ *n* = 29). Single-cell plots are shown in Supplemental Figure 14. In all panels, time point zero indicates the first detection of PI/Sytox Green. All scale bars, 10 µm

LPS transfection is a non-canonical inflammasome stimulus that triggers caspase-11- and GSDMD-mediated pyroptosis, but subcellular events during caspase-11-induced pyroptosis have not been defined [5, 8]. LPS transfection-induced pyroptosis was associated with a robust Fluo4 signal in wild-type macrophages, indicating that early Ca^2+^ influx is a shared commonality of pyroptosis induction by the non-canonical and canonical inflammasome pathways (Fig. 8a). LPS transfection also resulted in a loss of TMRM signal and mitochondrial decay prior to cell lysis (Fig. 8b), akin to our observations with the canonical inflammasome agonists LeTx and FlaTox (Fig. 3). Having established that pyroptosis induction by the non-canonical inflammasome shares Ca^2+^ influx and mitochondrial decay as early features with pyroptosis induced through canonical inflammasome pathways, we next addressed the role of GSDMD in these processes. Contrary to canonical inflammasome agonists LeTx and FlaTox that triggered fast induction of apoptosis in *GSDMD*^*-/-*^ macrophages (Supplemental Fig. 12), incorporation of PI by LPS-transfected *GSDMD*^*-/-*^ macrophages was delayed by approximately 5 h relative to pyroptotic wild-type macrophages (Supplemental Fig. 13), thus providing a suitable time window for examining the potential role of GSDMD in pyroptosis-associated early Ca^2+^ influx and mitochondrial decay. Notably, GSDMD deficiency not only protected against pyroptotic plasma membrane rupture during the timeframe of imaging, but also abolished the early Ca^2+^ influx seen in LPS-transfected wild-type macrophages (Fig. 8a, c, d, Supplemental Fig. 14a and Supplemental Movie 18). LPS-transfected *GSDMD*^*-/-*^ macrophages also maintained their mitochondrial polarization during the imaging timeframe, unlike LPS-transfected wild-type macrophages that lost their mitochondrial membrane potential during pyroptosis induction (Fig. 8b, e, f, Supplemental Fig. 14b and Supplemental Movie 19). To conclude, we show that both early Ca^2+^ influx and mitochondrial decay are conserved mechanisms of pyroptotic cell demise that are mediated by GSDMD and precede plasma membrane rupture following caspase-11 activation.

### Pyroptosis causes non-selective extracellular release of cytosolic and organellar proteins

**Fig. 9:**
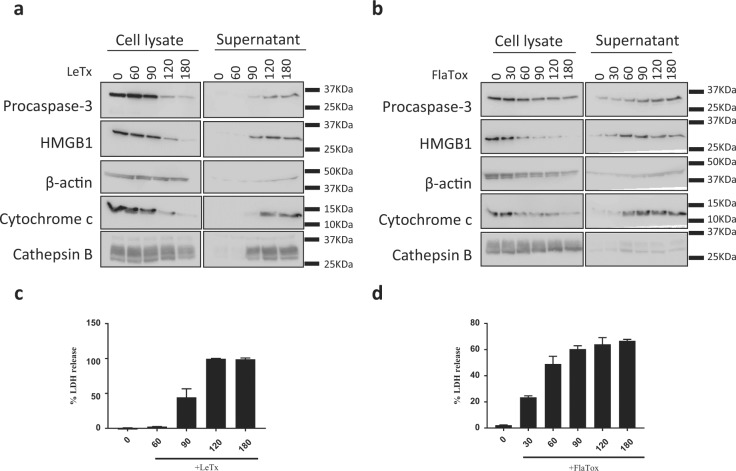
Pyroptosis triggers non-selective release of cytosolic and organellar proteins. Culture supernatants and cell lysates of B6^Nlrp1b+^ BMDMs stimulated with LeTx (**a**) or FlaTox (**b**) for the depicted durations were analyzed by western blotting for the indicated proteins. **c**,** d** Culture supernatants used in (**a, b**) were assayed for LDH activity. Data are representative of 3 independent experiments

The extracellular release of host-derived damage-associated molecular patterns (DAMPs) that are normally kept in the intracellular milieu is considered an important mechanism by which necrotic cell death promotes inflammation. In addition to IL-1β and IL-18, pyroptotic cells are known to release the cytosolic protein LDH—the enzymatic activity of which in culture media is often used as a surrogate marker of plasma membrane rupture—and the nuclear DAMPs HMGB1 and IL-1α [3, 33]. Pyroptotic cells were recently shown to contain organelles and microbial pathogens inside the ‘pore-induced intracellular trap’ [34], but whether proteins that reside in membrane-bound organelles are spilled extracellularly is unclear. To address this question, we stimulated B6^Nlrp1b+^ macrophages with LeTx or FlaTox for the indicated durations (Fig. 9a, b) before the extracellular medium and total cell lysates were analyzed by western blotting for a suite of organellar proteins. In parallel, the extracellular medium was analyzed for LDH activity, the near-maximal values of which were reached 120 and 90 min post treatment for LeTx- and FlaTox-stimulated cells, respectively (Fig. 9c, d). The extracellular levels of procaspase-3 raised concurrent herewith, whereas the cellular pool of this cytosolic protein diminished accordingly over time (Fig. 9c, d). β-Actin and the nuclear DAMP HMGB1 were also retrieved in supernatants of both LeTx- and FlaTox-stimulated cells. Moreover, mitochondrial cytochrome *c* and lysosomal cathepsin B were spilled with comparable kinetics in the extracellular space (Fig. 9a, b), consistent with our observation that both mitochondria and lysosomes are damaged during pyroptosis. Together, these results highlight that a broad set of proteins and potential DAMPs originating from damaged organelles and the cytosol alike are targeted for non-selective extracellular release during pyroptosis.

## Discussion

The requirement for the proteolytic activity of inflammatory caspases fundamentally distinguishes pyroptosis from apoptosis, necroptosis and accidental necrosis. It has long been recognized that the induction of pyroptosis in parallel to caspase-1-mediated maturation of IL-1β and IL-18 may constitute a powerful defense mechanism of the host against microbial pathogens [1, 25, 34–36]. Despite its evident role in anti-microbial host defense and the recent identification of GSDMD as a key pyroptosis effector molecule, the distinguishing features and subcellular dynamics of pyroptotic cells have remained largely unmapped. Our single-cell analysis of pyroptosis kinetics revealed that pyroptosis is characterized by ionic fluxing and cell swelling that is accompanied by mitochondrial depolarization and lysosome leakage well before cells ultimately lost their plasma membrane integrity concomitant with evidence of late-stage nuclear condensation. We thus charted a conserved sequence of subcellular events that preceded plasma membrane rupture by up to 20 min, challenging the current model that pyroptosis is induced by the insertion in the plasma membrane of pre-assembled non-selective GSDMD pores with inner diameters of 10–20 nm. Pores of this size would likely maintain osmolarity by allowing the simultaneous passage of ions, small molecules and proteins, and thus would not be able to account for the early cell swelling we observed. Important in this regard is our demonstration that Ca^2+^ influx precedes the uptake of cell-impermeant fluorescent dye molecules (sized 400 Da or more), and that entry of the latter proceeds sequentially according to their *M*_w_, suggesting that pyroptosis is executed by an increasing permeability of the plasma membrane and that formation of large non-selective GSDMD pores is a late-stage pyroptotic event that is closely associated with the breakdown of the plasma membrane. Consistently, an early estimate of the pyroptotic membrane pore diameter in *S*. Typhimurium-infected macrophages suggested it to be 1.1–2.4 nm in diameter, although the identity of such pores has not been defined [26]. Considering our demonstration that GSDMD is required for early Ca^2+^ influx and mitochondrial membrane depolarization of LPS-transfected macrophages, it is tempting to speculate that GSDMD_N_ monomers insert in membranes individually or as small oligomers that further assemble into higher order oligomers, reminiscent of the mechanism used by Bax in the mitochondrial outer membrane and cation-selective actinoporin pores [37]. Such ‘non-concerted’ membrane insertion model for GSDMD_N_ pore assembly would account for both early ion-selective fluxing and late-stage non-selective GSDMD_N_ pore formation. Indeed, GSDMD_N_ oligomers formed in GSDMD_N_-overexpressing 293T cells were reported to be heterogeneous in size [32].

We further observed that pyroptotic plasma membrane rupture is kinetically closely associated with Annexin-V positivity. Considering that phosphatidylserine is an ‘eat-me’ signal for phagocytes, it will be interesting to determine the timeframe by which extracellular release of IL1β and IL-18 precedes efferocytosis of the pyroptotic corpse, and to study the role of phosphatidylserine in this process. We also showed that pyroptosis is associated with an apparently non-discriminatory extracellular spilling of proteins from both the cytosolic and organellar compartments. The latter suggests that enzymes and DAMPs that normally reside in membrane-bound organelles such as the nucleus, mitochondria and lysosomes might potentially exert specific roles and contribute to an inflammatory milieu following their release from pyroptotic cells. In conclusion, this study charted a chronological sequence of subcellular events that define pyroptotic cell death at the single-cell level, and provides a dynamic framework for understanding the role of GSDMD during pyroptosis.

## Methods

### Mice

B6^Nlrp1b+^[13], H2K-Bcl2^Tg^ [38] and GSDMD^-/-^ [5] mice have been reported. C57BL/6J mice were originally bought from the Jackson Laboratories and bred in-house. Mice were housed in individually ventilated cages and kept under pathogen-free conditions at the animal facilities of Ghent University. All animal experiments were conducted with permission of the ethics committee on laboratory animal welfare of Ghent University.

### Reagents

Recombinant expression and purification of LFn-FlaA was performed as previously described [19]. *B. anthracis* protective antigen (PA) and lethal factor (LF) were acquired from List Biologicals. TMRM (T668), Lysotracker (L7528), Sytox Green (S7020), Mitotracker (M7512), Fluo4 (F14217), Pluronic F-127 (P6867), Hoechst 33342 Trihydrochloride Trihydrate (H1399), PI (P3566) and CTB coupled to Alexa 594 (C22842) or 647 (C34778) were purchased from Thermo Scientific. PI solution (556463) and Annexin-V–FITC (556419) were from BD Biosciences. The antibodies used in the study were anti-Caspase-1 (AG-20B-0042-C10, Adipogen), anti-Cathepsin B (31718S, Cell Signaling Technology), anti-Cytochrome *c* (11940S, Cell Signaling Technology), anti-HMGB1 (ab18256, Abcam), anti-Caspase-3 (9662S, Cell Signaling Technology) and anti-β-Actin-HRP (sc-47778, Santa Cruz Biotechnology), anti-BID (AF860, R&D Systems). Horseradish peroxidase (HRP)-conjugated secondary antibodies were acquired from Jackson Immunoresearch Laboratories and enhanced chemiluminescence solution was from Thermo Scientific. Punicalagin (P0023) was from Sigma Aldrich and the CytoTox 96 Non-Radioactive Cytotoxicity Assay (G1780) and FugeneHD Transfection Reagent were purchased from Promega. Y27632 and (−)-blebbistatin were acquired from Selleckchem. Pam3csk4 (tlrl-pms) and LPS-SM (tlrl-smlps) were acquired from Invivogen.

### Macrophage differentiation and stimulation

Macrophages were differentiated by culturing bone marrow progenitor cells in Iscove’s modified Dulbecco’s medium (IMDM; Lonza) containing 10% (v/v) heat-inactivated fetal bovine serum (FBS), 30% (v/v) L929 cell-conditioned medium, 1% (v/v) non-essential amino acids (Lonza), 100 U/ml penicillin and 100 mg/ml streptomycin at 37 °C in a humidified atmosphere containing 5% CO_2_ for 6 days. BMDMs were then seeded into 8-well µ-slides (Ibidi) or in multiple wells plates as needed, in IMDM containing 10% FBS, 1% non-essential amino acids and antibiotics. For NLRP1b inflammasome activation, cells were stimulated with LeTx (1 µg/ml PA combined with 0.5 or 1 µg/ml LF). The NLRC4 inflammasome was activated in BMDMs treated with FlaTox (1 µg/ml PA combined with 1 µg/ml LFn-FlaA). Necroptosis was induced with TNF (20 ng/ml), BV6 (2 µM) and zVAD-fmk (50 µM). In some experiments, BMDMs were treated with Y27632 (10 µM), (−)-blebbistatin (10 µM) or punicalagin (25 or 50 µM) before inflammasome stimuli. For the non-canonical inflammasome activation, BMDMs were primed with Pam3csk4 (1 µg/ml) for 6 h in Opti-MEM medium. Then, cells were either mock treated or stimulated with 0.25% (v/v) Fugene with or without LPS (2 µg/ml).

### Live-cell imaging

BMDMs were incubated with TMRM (400 nM), Lysotracker (50 nM), Mitotracker (25 nM) and Hoechst (20 ng/ml) for 30 min at 37 °C, after which they were washed to fresh culture media. For plasma membrane labeling, cells were incubated with CTB Alexa 594 or CTB Alexa 647 (10 µg/ml) for 30 min at 4 °C and then washed with fresh media. In other experiments, Fluo4 (5 µM) was mixed in a 1:1 (v/v) ratio with Pluronic F-127 (20% w/v in dimethyl sulfoxide) before adding the mixture to cells in Hanks' balanced salt solution. Cells were incubated with Fluo4 solution for 30 min at room temperature and washed to fresh media containing Fluo4 (2.5 µM). Imaging was performed in culture media containing Sytox Green (10 nM) or PI (0.5 µg/ml).

### Image acquisition and processing

In each experiment, 2–3 fields/condition were selected for time-lapse imaging using an observer Z.1 spinning disk microscope (Zeiss, Zaventem, Belgium) equipped with a Yokogawa disk CSU-X1. Cells were incubated in a chamber with a 5% CO_2_ atmosphere at 37 °C throughout the experiment. Differential interference contrast (DIC) and fluorescence images were acquired at regular intervals ranging from 1 to 10 min apart, with the use of a pln Apo 40×/1.4 oil DIC III objective and a Rolera em-c2 camera. Image acquisition started at the moment of stimulation with LeTx, FlaTox and TBz; and after 2 h of LPS transfection for pyroptosis induction by the non-canonical inflammasome pathway. Cells were imaged at least until they became positive for Sytox Green or PI. *Z*-stacks consisted of 5 or 8 planes with a *Z*-interval of 2 µm. Mock-treated cells were imaged in parallel to ensure that imaging and staining procedures were not cytotoxic.

Representative images and movies were extracted and edited in Fiji software. Image quantifications were performed on the three-dimensional images through voxel-based quantification of the mean fluorescence intensity (MFI), volume and sphericity in Imaris x64 7.7.2 (Bitplane) software package. Obtained fluorescence data were normalized for bleaching against the corresponding time points of mock-treated cells that were imaged in parallel. Cell volume and MFI percentages were plotted relative to each cell’s initial value, except for Annexin-V, Sytox Green and PI percentages, which were plotted against the maximum noted values. The Feret diameter of Hoechst-stained nuclei was determined for each *Z*-slice using Fiji software, and the highest value obtained for each time point was retained and shown. All graph data on main figures depict the mean of multiple cells that were quantified in each independent experiment ± SD. All graph data on supplementary figures depict the individual values obtained for each cell after normalization. The time indication in presented graphs and image panels is relative to the moment when cells became positive for Sytox Green or PI. Time zero in movie stills refers to the start of imaging.

### Western blotting

Unless otherwise stated, cell lysates and culture supernatants were combined for western blotting. Protein samples were denatured in Laemmli buffer, boiled at 95 °C for 10 min, separated by sodium dodecyl sulfate–polyacrylamide gel electrophoresis and transferred to polyvinylidene difluoride membranes. Phosphate-buffered saline (PBS) supplemented with 0.05% Tween-20 (v/v) and 3% nonfat dry milk (w/v) was used for blocking and washing of membranes. Immunoblots were incubated overnight with primary antibodies against cathepsin B and cytochrome *c* (1:1000 in Tris-buffered saline, 0.1% Tween-20, 5% BSA), HMGB1 or caspase-3 (1:1000 in PBS, 0.1% Tween-20, 5% nonfat dry milk), caspase-1 or BID (1:1000 in PBS, 0.05% Tween-20, 3% nonfat dry milk), followed by HRP-conjugated secondary antibodies raised against mouse, goat or rabbit (1:5000). The β-Actin-HRP antibody was used at 1:5000 in PBS 0.1% Tween-20, 5% nonfat dry milk. All proteins were detected by enhanced chemiluminescence.

### Kinetic of LDH release

The supernatant of cells stimulated at various time points was collected and centrifuged at 300 × *g* for 5 min to remove cellular debris. LDH measurement was performed with the CytoTox 96 Non-Radioactive Cytotoxicity Assay kit according to the manufacturers’ instructions, in samples diluted 1:5 in PBS. Data were plotted considering the O.D. value obtained in a well treated with Triton X-100 as 100%.

### Cell permeabilization kinetics (Incucyte)

BMDMs were plated and stimulated in a 96-well plate in media containing PI (0.1 µg/ml) and data were acquired with a 10× objective using the IncuCyte Zoom system (Essen BioScience) in a CO_2_ and temperature-controlled environment. Each condition was run in (technical) duplicate. The number of fluorescent objects was counted with Incucyte ZOOM (Essen BioScience) software and was plotted considering as 100% the highest value obtained in a well treated with Triton X-100.

## Electronic supplementary material

Supplementary figures 1-14

Supplementary Data

Supplemental Movie 1

Supplemental Movie 2

Supplemental Movie 3

Supplemental Movie 4

Supplemental Movie 5

Supplemental Movie 6

Supplemental Movie 7

Supplemental Movie 8

Supplemental Movie 9

Supplemental Movie 10

Supplemental Movie 11

Supplemental Movie 12

Supplemental Movie 13

Supplemental Movie 14

Supplemental Movie 15

Supplemental Movie 16

Supplemental Movie 17

Supplemental Movie 18

Supplemental Movie 19
