# Supplementary material for: Single-cell analysis of pyroptosis dynamics reveals conserved GSDMD-mediated subcellular events that precede plasma membrane rupture
Source: Cell Death Differ. 2018 Apr 17;26(1):146–61. doi: 10.1038/s41418-018-0106-7 (PMC6294780; doi:10.1038/s41418-018-0106-7)
Supplement: Supplementary file 1 — Supplementary figures 1-14 [file 41418_2018_106_MOESM1_ESM.pdf]

Supplementary Data for:

Single-cell analysis of pyroptosis dynamics reveals conserved GSDMD-mediated subcellular events that precede plasma membrane rupture

Nathalia M. de Vasconcelos[1,2], Nina Van Opdenbosch[1,2], Hanne Van Gorp[1,2], Eef Parthoens[2,3], Mohamed Lamkanfi[1,2,4]

[1] *Department of Internal Medicine, Ghent University, Ghent, B-9000, Belgium*

[2] *VIB-UGhent Center for Inflammation Research, VIB, Ghent, B-9000, Belgium*

[3] *VIB Bioimaging Core, VIB, Ghent, B-9000, Belgium*

[4] *Current address: Janssen Immunosciences, World Without Disease Accelerator, Janssen Pharmaceutical Companies of Johnson & Johnson, Beerse, B-2340, Belgium*

Supplementary Figure Legends

Supplementary Movie Legends

Supplementary Figures 1-14

Supplementary Movies 1-19

Supplementary Figure Legends

Supplemental Figure 1. PS exposure during pyroptosis. **a, b** B6$^{Nlrp1b+}$ BMDMs were stimulated with LeTx (**a**) or FlaTox (**b**) and imaged in culture media containing Annexin-V-FITC and PI. Confocal images were acquired every 3 minutes. Graphs show the percentage of mean fluorescence intensity (MFI) of single cells (LeTx n=18; FlaTox n=21), calculated as described in *Methods*, of Annexin-V (upper panel) or PI (lower panel) signals. In all panels time point zero indicates the first detection of PI. Relates to Figure 2b, d.

Supplemental Figure 2. Mitochondrial morphology of mock-treated BMDMs. B6$^{Nlrp1b+}$ BMDMs preloaded with Mitotracker Red CMXRos were imaged in culture media containing Sytox Green (n=50). A single plane of a representative cell is shown. Scale bars, 10 µm.

Supplemental Figure 3. Mitochondrial damage during pyroptosis. a, b B6$^{Nlrp1b+}$ BMDMs were preloaded with TMRM and stimulated with either LeTx (**a**) or FlaTox (**b**) and imaged in culture media containing Sytox Green. Confocal images were acquired every 3 minutes. Graphs show the percentage of mean fluorescence intensity (MFI) of single cells (LeTx, n=28; FlaTox n=28), calculated as described in *Methods*, of TMRM (upper panel) or Sytox Green (lower panel) signals. "Mock" lines represent the average of the values obtained in unstimulated cells, imaged in parallel. In all panels time point zero indicates the first detection of Sytox Green. Relates to Figure 3c, e.

Supplemental Figure 4. Bax/Bak pores are dispensable for pyroptosis-associated mitochondrial damage. a Protein lysates of B6$^{Nlrp1b+}$ BMDMs stimulated with LeTx for 60, 120 or 180 minutes were analyzed by Western blotting for BID. **a-c**, Protein lysate of B6$^{Nlrp1b+}$ and B6$^{Nlrp1b+}$H2K-Bcl2^{Tg} BMDMs that have been stimulated with LeTx for 90 or 180 minutes were assayed by Western blotting for caspase-1 maturation (**b**), and their culture supernatants were assayed for LDH activity (**c**). **d, e** B6$^{Nlrp1b+}$ and B6$^{Nlrp1b+}$H2K-Bcl2^{Tg} BMDMs were loaded with TMRM and stimulated with LeTx in culture media containing Sytox Green. Confocal images were acquired every 3 minutes. Graphs show the percentage of mean fluorescence intensity (MFI) calculated as described in *Methods*, and values represent the mean ± SD of individual cells imaged in 3 independent experiments (B6$^{Nlrp1b+}$ n=26; B6$^{Nlrp1b+}$H2K-Bcl2^{Tg} n=21). Fluorescent micrographs show the maximum intensity projection of a representative cell. In panels **d, e** time point zero indicates the first detection of Sytox Green. All scale bars, 10 µm.

Supplemental Figure 5. Lysosomes decay prior to pyroptotic cell lysis. a, b B6$^{Nlrp1b+}$ or B6 BMDMs preloaded with Lysotracker and stimulated with LeTx (**a**) or FlaTox (**b**), respectively, were imaged throughout cell death in culture media containing Sytox Green. Confocal images were taken every 3 minutes. Graphs show the percentage of mean fluorescence intensity (MFI) of single cells (LeTx, n=27; FlaTox n=19), calculated as described in *Methods*, of Lysotracker (upper panel) or Sytox Green (lower panel) signals. "Mock" lines represent the average of the values obtained in unstimulated cells, imaged in parallel. In all panels time point zero indicates the first detection of Sytox Green. Relates to Figure 4b, d.

Supplemental Figure 6. Nuclei round up and condense during pyroptosis. a-d B6$^{Nlrp1b+}$ BMDMs were preloaded with Hoechst dye and stimulated with LeTx (**a, b**) or FlaTox (**c, d**) before imaging in culture media containing Sytox Green. Confocal images were acquired every 10 minutes. Graphs show values for nuclear sphericity (**a, c**, upper panels) or Feret's diameter (**b, d**, upper panels) based on Hoechst staining or the percentage of mean fluorescence intensity (MFI) of Sytox Green signal (lower panels) of single cells (LeTx: Sphericity n=24, Feret's diameter n= 18; FlaTox: Sphericity n=26, Feret's diameter n=20), calculated as described in *Methods*. "Mock" lines represent the average of the values obtained in unstimulated cells, imaged in parallel. In all panels time point zero indicates the first detection of Sytox Green. Relates to Figure 5b, c, d, e.

Supplemental Figure 7. Cell swelling precedes pyroptotic cell rupture. a, b B6$^{Nlrp1b+}$ or B6 BMDMs stained with Cholera Toxin subunit B-Alexa 594 (rCTB) were stimulated with LeTx (**a**) or FlaTox (**b**), respectively, and imaged in culture media containing Sytox Green. Confocal images were acquired every 1.5 minutes. Graphs show the percentage of cell volume quantifications based on rCTB-Alexa 594 staining (upper panel) or the mean fluorescence intensity (MFI) of Sytox Green signal (lower panels) of single cells (LeTx, n=26; FlaTox n=16), calculated as described in *Methods*. "Mock" lines represent the average of the values obtained in unstimulated cells, imaged in parallel. In all panels time point zero indicates the first detection of Sytox Green. Relates to Figure 6b, d.

Supplemental Figure 8. Punicalagin inhibits LeTx-induced pyroptosis caspase-1 activation. a, b B6$^{Nlrp1b+}$ BMDMs were pretreated with Punicalagin at the indicated concentrations (µM) and stimulated with LeTx for 90 min. Culture supernatants were assayed for LDH (**a**), and protein lysates for caspase-1 by Western blotting (**b**). (**c, d**) BMDMs were pretreated with Punicalagin (50 µM) and stimulated with LeTx for 90 or

180 minutes. Culture supernatants were assayed for LDH (**c**), and protein lysates for caspase-1 by Western blotting (**d**). (**e, f**) B6$^{Nlrp1b+}$ BMDMs that had been preloaded with the Ca^{2+} indicator Fluo4 and stained with CTB-Alexa 647 (rCTB) were incubated with Punicalagin (50 µM) or vehicle control before cells were stimulated with LeTx and imaged in culture media containing PI. Confocal images were acquired every 1.5 minutes. Graphs show the percentage of mean fluorescence intensity (MFI) of PI (upper panel, left axis) and Fluo4 (lower panel) and cell volume quantifications based on rCTB-Alexa 647 staining (upper panel, right axis), all calculated as described in *Methods*. Values represent the mean ± SD of 2 independent experiments (LeTx n=11; Punicalagin+LeTx n=9). Fluorescent micrographs show the maximum intensity projection (PI and Fluo4) or the single plane (rCTB) of a representative cell. All scale bars, 10 µm.

Supplemental Figure 9. Punicalagin inhibits FlaTox-induced caspase-1 activation. a, b B6$^{Nlrp1b+}$ BMDMs were pretreated with Punicalagin at the indicated concentrations (µM) and stimulated with FlaTox for 30 min. Culture supernatants were assayed for LDH (**a**), and protein lysates for caspase-1 by Western blotting (**b**). BMDMs were pretreated with Punicalagin (50 µM) and stimulated with FlaTox for 30 or 60 minutes. Culture supernatants were assayed for LDH (**c**), and protein lysates for caspase-1 by Western blotting (**d**). Data are representative of 2 independent experiments.

Supplemental Figure 10. Pyroptotic Ca^{2+} influx occurs prior to total membrane permeabilization. **a-d** B6$^{Nlrp1b+}$ BMDMs preloaded with the cell-permeant Ca^{2+} indicator Fluo4 were imaged after stimulation with LeTx (**a**) or FlaTox (**b**) in culture media containing PI. Confocal images were acquired every 1.5 minutes. Graphs show the percentage of mean fluorescence intensity (MFI) of single cells (LeTx, n=24; FlaTox n=23), calculated as described in *Methods*, of Fluo4 (upper panel) or PI (lower panel) signals. “Mock” lines represent the average of the values obtained in unstimulated cells, imaged in parallel. In all panels time point zero indicates the first detection of PI. Relates to Figure 7b, d.

Supplemental Figure 11. Pyroptotic cells provide sequential permeability to Ethidium Bromide and Sytox Green. a, b B6$^{Nlrp1b+}$ BMDMs were stimulated with LeTx and imaged in culture media containing Ethidium Bromide and Sytox Green. Confocal images were acquired every 1 minute. Graph shows the percentage of mean fluorescence intensity (MFI) calculated as described in *Methods*, and values represent the mean ± SD of individual cells imaged in 3 independent experiments (n=50). Fluorescent micrographs show the maximum intensity projection of a representative cell. In all panels time point zero indicates the first detection of Sytox Green. All scale bars, 10 µm.

Supplemental Figure 12. Canonical inflammasome stimuli induce apoptosis in GSDMD-deficient macrophages with kinetics similar to pyroptosis induction in WT cells. a, b BMDMs of indicated genotypes were stimulated with LeTx (**a**) or FlaTox (**b**). Images show the bright field of representative cells (LeTx n=20; FlaTox n=18). All scale bars, 10 µm.

Supplemental Figure 13. Delayed membrane permeabilization following non-canonical inflammasome activation in GSDMD-deficient macrophages. Pam3csk4-primed GSDMD-deficient and –sufficient B6 BMDMs were transfected with LPS (2 µg/ml, Fugene+LPS), treated with Fugene alone or kept without treatment and imaged in media containing PI. The number of positive cells was quantified relative to a Triton-x100-treated well (considered 100%) of each genotype. Values represent mean ± SD of technical duplicates of a representative experiment out of 3 independent experiments.

Supplemental Figure 14. GSDMD deficiency recues Ca^{2+} influx and mitochondrial decay associated with activation of the non-canonical inflammasome. a, b Pam3csk4-primed BMDMs of WT (**a**) and GSDMD$^{-/-}$ (**b**) mice were preloaded with the cell-permeant Ca^{2+} indicator Fluo4 and imaged after transfection with LPS (2 µg/ml, Fugene+LPS), Fugene alone or mock-treated in culture media containing PI. Confocal images were acquired every 2 minutes. **c, d** BMDMs of WT (**c**) and GSDMD$^{-/-}$ (**d**) mice were preloaded with TMRM and imaged after transfection with LPS (2 µg/ml, Fugene+LPS), Fugene alone or mock-treated in culture media containing Sytox Green. Confocal images were acquired every 3 minutes. Graphs show the percentage of mean fluorescence intensity (MFI) of single cells (Fluo4: WT n=18, GSDMD$^{-/-}$ n=28; TMRM: WT n=18, GSDMD$^{-/-}$ n=29), calculated as described in *Methods*, of Fluo4 and TMRM (upper panels) or PI and Sytox Green (lower panels) signals. "Mock" lines represent the average of the values obtained in unstimulated cells that were imaged in parallel. In all panels time point zero indicates the first detection of PI. Relates to Figure 8d, f.

Supplementary Movie Legends

Supplemental Movie 1. Necroptotic cells detach and round up before becoming Sytox Green-positive. B6$^{Nlrp1b+}$ BMDMs were stimulated with TNF+BV6+zVAD-fmk (20 ng/ml, 2 µM and 50 µM, respectively) and imaged in culture media containing Sytox Green. Confocal images were acquired every three minutes. Fluorescent micrographs show the maximum intensity projection (Sytox Green) of a representative cell

from 30 analysed cells in 4 independent experiments. Time point zero indicates the start of imaging. Relates to Figure 1a. Scale bar, 10 µm.

Supplemental Movie 2. Pyroptotic cells remain attached during membrane permeabilization. Cholera Toxin subunit B-Alexa 647 (rCTB)-stained B6$^{Nlrp1b+}$ BMDMs were stimulated with LeTx and imaged in culture media containing Sytox Green. Confocal images were acquired every 3 minutes. Fluorescent micrographs show the maximum intensity projection (Sytox Green) or the single plane (rCTB) of a representative cell from 30 analysed cells in 3 independent experiments. Time point zero indicates the start of imaging. Relates to Figure 1b. Scale bar, 10 µm.

Supplemental Movie 3. Phosphatidylserine exposure during LeTx-induced pyroptosis. B6$^{Nlrp1b+}$ BMDMs were stimulated with LeTx and imaged in culture media containing Annexin-V-FITC and PI. Confocal images were acquired every 3 minutes. Fluorescent micrographs show the maximum intensity projection of a representative cell from 18 analysed cells in 3 independent experiments. Time point zero indicates the start of imaging. Relates to Figure 2a. Scale bar, 10 µm.

Supplemental Movie 4. Mock-treated BMDMs are negative for Annexin-V and PI staining during imaging. Mock-treated B6$^{Nlrp1b+}$ BMDMs were imaged in culture media containing Annexin-V-FITC and PI. Confocal images were acquired every 3 minutes. Fluorescent micrographs show the maximum intensity projection of a representative cell from 25 analysed cells in 3 independent experiments. Time point zero indicates the start of imaging. Relates to Figure 2. Scale bar, 10 µm.

Supplemental Movie 5. Phosphatidylserine exposure is a late-stage event during FlaTox-induced pyroptosis. B6$^{Nlrp1b+}$ BMDMs were stimulated with FlaTox and imaged in culture media containing Annexin-V-FITC and PI. Confocal images were acquired every 3 minutes. Fluorescent micrographs show the maximum intensity projection of a representative cell from 21 analysed cells in 3 independent experiments. Time point zero indicates the start of imaging. Relates to Figure 2c. Scale bar, 10 µm.

Supplemental Movie 6. Mitochondrial morphology changes during LeTx-induced pyroptosis. B6$^{Nlrp1b+}$ BMDMs preloaded with Mitotracker Red CMXRos were mock- (upper panel) or LeTx-stimulated (lower panel) and imaged in culture media containing Sytox Green. Confocal images were acquired every 3 minutes. Fluorescent micrographs show the maximum intensity projection (Sytox Green) or the single plane (Mitotracker) of a representative cell from 50 analysed cells in 3 independent experiments. Time point zero indicates the start of imaging. Relates to Figure 3a. Scale bar, 10 µm.

Supplemental Movie 7. Mitochondria decay during pyroptosis in LeTx-stimulated cells. $B6^{Nlrp1b+}$ BMDMs preloaded with TMRM were mock- (upper panel) or LeTx-treated (lower panel) and imaged in culture media containing Sytox Green. Confocal images were acquired every 3 minutes. Fluorescent micrographs show the maximum intensity projection of a representative cell from 28 analysed cells in 5 independent experiments. Time point zero indicates the start of imaging. Relates to Figure 3b. Scale bar, 10 µm.

Supplemental Movie 8. Mitochondrial decay during FlaTox-induced pyroptosis. $B6^{Nlrp1b+}$ BMDMs preloaded with TMRM were either mock (upper panel) or FlaTox-treated (lower panel) and imaged in culture media containing Sytox Green. Confocal images were acquired every 3 minutes. Fluorescent micrographs show the maximum intensity projection of a representative cell from 28 analysed cells in 5 independent experiments. Time point zero indicates the start of imaging. Relates to Figure 3d. Scale bar, 10 µm.

Supplemental Movie 9. Lysosomes decay during pyroptosis triggered by LeTx. $B6^{Nlrp1b+}$ BMDMs preloaded with Lysotracker were mock- (upper panel) or LeTx-treated (lower panel) and imaged in culture media containing Sytox Green. Confocal images were taken every 3 minutes. Fluorescent micrographs show the maximum intensity projection of a representative cell from 27 analysed cells in 3 independent experiments. Time point zero indicates the start of imaging. Relates to Figure 4a. Scale bar, 10 µm.

Supplemental Movie 10. FlaTox-induced pyroptosis is associated with lysosomal decay. B6 BMDMs preloaded with Lysotracker were mock- (upper panel) or FlaTox-treated (lower panel) and imaged in culture media containing Sytox Green. Confocal images were taken every 3 minutes. Fluorescent micrographs show the maximum intensity projection of a representative cell from 19 analysed cells in 3 independent experiments. Time point zero indicates the start of imaging. Relates to Figure 4c. Scale bar, 10 µm.

Supplemental Movie 11. Nuclear morphology changes during LeTx-induced pyroptosis. $B6^{Nlrp1b+}$ BMDMs preloaded with Hoechst were mock- (upper panel) or LeTx-treated (lower panel) before being imaged in culture media containing Sytox Green. Confocal images were acquired every 10 minutes. Fluorescent micrographs show the maximum intensity projection of a representative cell from 24 analysed cells in 3 independent experiments. Time point zero indicates the start of imaging. Relates to Figure 5a. Scale bar, 10 µm.

Supplemental Movie 12. Nuclear alterations during FlaTox-triggered pyroptosis. $B6^{Nlrp1b+}$ BMDMs preloaded with Hoechst were mock- (upper panel) or FlaTox-treated (lower panel) before being imaged

in culture media containing Sytox Green. Confocal images were acquired every 10 minutes. Fluorescent micrographs show the maximum intensity projection of a representative cell from 26 analysed cells in 3 independent experiments. Time point zero indicates the start of imaging. Relates to Figure 5c. Scale bar, 10 µm.

Supplemental Movie 13. Cell volume increases in LeTx-triggered pyroptosis. $B6^{Nlrp1b+}$ BMDMs stained with Cholera Toxin subunit B-Alexa 594 (rCTB) were mock- (upper panel) or LeTx-treated (lower panel) and imaged in culture media containing Sytox Green. Confocal images were acquired every 1.5 minutes. Fluorescent micrographs show the maximum intensity projection (Sytox Green) or the single plane (rCTB) of a representative cell out from 26 analysed cells in 3 independent experiments. Time point zero indicates the start of imaging. Relates to Figure 4a. Scale bar, 10 µm.

Supplemental Movie 14. Cellular swelling during FlaTox-mediated pyroptosis. B6 BMDMs stained with Cholera Toxin subunit B-Alexa 594 (rCTB) were mock- (upper panel) or FlaTox-treated (lower panel) and imaged in culture media containing Sytox Green. Confocal images were acquired every 1.5 minutes. Fluorescent micrographs show the maximum intensity projection (Sytox Green) or the single plane (rCTB) of a representative cell from 16 analysed cells in 3 independent experiments. Time point zero indicates the start of imaging. Relates to Figure 4c. Scale bar, 10 µm.

Supplemental Movie 15. Ca^{2+} influx occurs during LeTx-induced pyroptosis prior to total membrane permeabilization. $B6^{Nlrp1b+}$ BMDMs preloaded with the cell-permeant Ca^{2+} indicator Fluo4 were mock- (upper panel) or LeTx-treated (lower panel) and imaged in culture media containing PI. Confocal images were acquired every 1.5 minutes. Fluorescent micrographs show the maximum intensity projection of a representative cell from 24 analysed cells in 4 independent experiments. Time point zero indicates the start of imaging. Relates to Figure 6a. Scale bar, 10 µm.

Supplemental Movie 16. FlaTox-induced pyroptosis features Ca^{2+} influx prior to total membrane rupture. $B6^{Nlrp1b+}$ BMDMs preloaded with the cell-permeant Ca^{2+} indicator Fluo4 were mock- (upper panel) or FlaTox-treated (lower panel) and imaged in culture media containing PI. Confocal images were acquired every 1.5 minutes. Fluorescent micrographs show the maximum intensity projection of a representative cell from 23 analysed cells in 4 independent experiments. Time point zero indicates the start of imaging. Relates to Figure 6c. Scale bar, 10 µm.

Supplemental Movie 17. Sequential incorporation of Ethidium Bromide and Sytox Green in pyroptotic cells. $B6^{Nlrp1b+}$ BMDMs were stimulated with LeTx and imaged in culture media containing Ethidium

Bromide and Sytox Green. Confocal images were acquired every 1 minute. Fluorescent micrographs show the maximum intensity projection of a representative cell from 50 analysed cells in 3 independent experiments. Time point zero indicates the start of imaging. Relates to Supplemental Figure 5a. Scale bar, 10 µm.

Supplemental Movie 18. GSDMD mediates early Ca^{2+} influx that precedes total membrane permeabilization following non-canonical inflammasome activation. Pam3csk4-primed BMDMs of WT (upper panel) or $GSDMD^{-/-}$ (lower panel) mice were preloaded with the cell-permeant Ca^{2+} indicator Fluo4 and transfected with LPS (2 µg/ml) for 90 minutes before imaging in culture media containing PI. Confocal images were acquired every 2 minutes. Fluorescent micrographs show the maximum intensity projection of a representative cell from 18 (WT) or 28 ($GSDMD^{-/-}$) analysed cells in 3 independent experiments. Time point zero indicates the start of imaging. Relates to Figure 8a, b. Scale bar, 10 µm.

Supplemental Movie 19. Lack of GSDMD rescues mitochondrial decay during pyroptosis in LPS-transfected cells. Pam3csk4-primed BMDMs of either WT (upper panel) or $GSDMD^{-/-}$ (lower panel) mice were preloaded with TMRM and transfected with LPS (2 µg/ml) for 90 minutes before imaging in culture media containing Sytox Green. Confocal images were acquired every 3 minutes. Fluorescent micrographs show the maximum intensity projection of a representative cell from 18 (WT) or 29 ($GSDMD^{-/-}$) analysed cells in 4 independent experiments of 12-18 imaged cells. Time point zero indicates the start of imaging. Relates to Figure 8d, e. Scale bar, 10 µm.

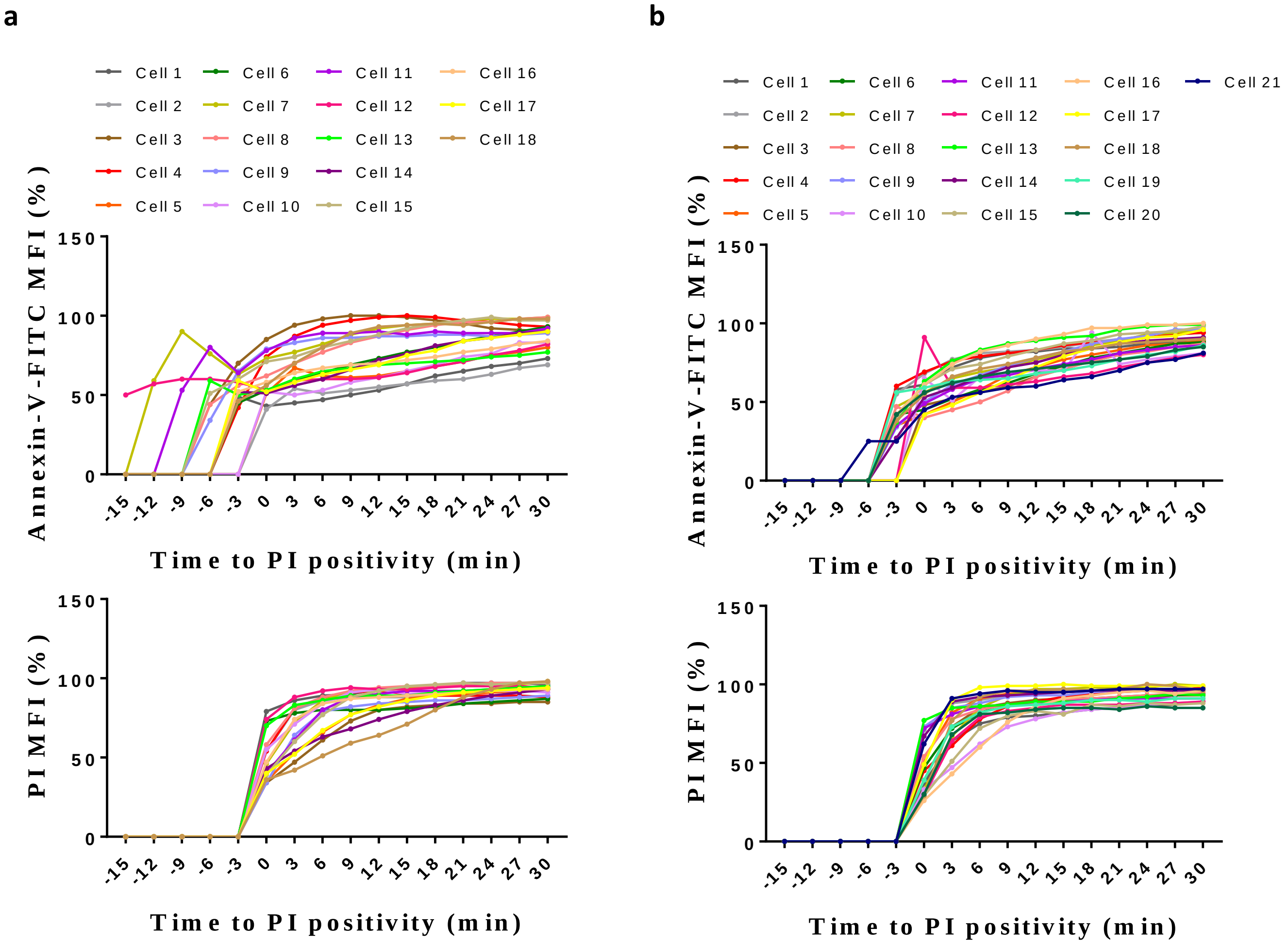
a
Cell 1
Cell 2
Cell 3
Cell 4
Cell 5
Cell 6
Cell 7
Cell 8
Cell 9
Cell 10
Cell 11
Cell 12
Cell 13
Cell 14
Cell 15
Cell 16
Cell 17
Cell 18
Annexin-V-FITC MFI (%)
150
100
50
0
-15
-12
-9
-6
-3
0
3
6
9
12
15
18
21
24
27
30
Time to PI positivity (min)
PI MFI (%)
150
100
50
0
-15
-12
-9
-6
-3
0
3
6
9
12
15
18
21
24
27
30
Time to PI positivity (min)
b
Cell 1
Cell 2
Cell 3
Cell 4
Cell 5
Cell 6
Cell 7
Cell 8
Cell 9
Cell 10
Cell 11
Cell 12
Cell 13
Cell 14
Cell 15
Cell 16
Cell 17
Cell 18
Cell 19
Cell 20
Cell 21
Annexin-V-FITC MFI (%)
150
100
50
0
-15
-12
-9
-6
-3
0
3
6
9
12
15
18
21
24
27
30
Time to PI positivity (min)
PI MFI (%)
150
100
50
0
-15
-12
-9
-6
-3
0
3
6
9
12
15
18
21
24
27
30
Time to PI positivity (min)

Suppl Fig 1

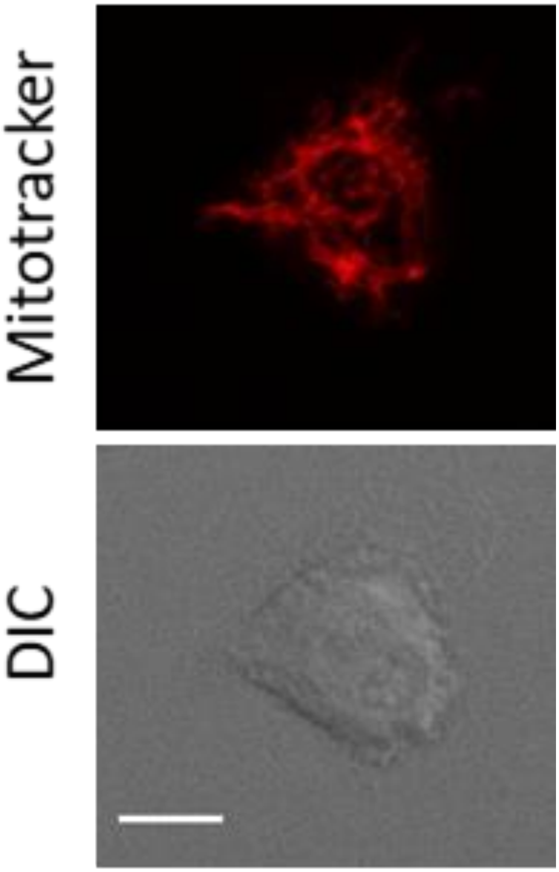

Suppl Fig 2

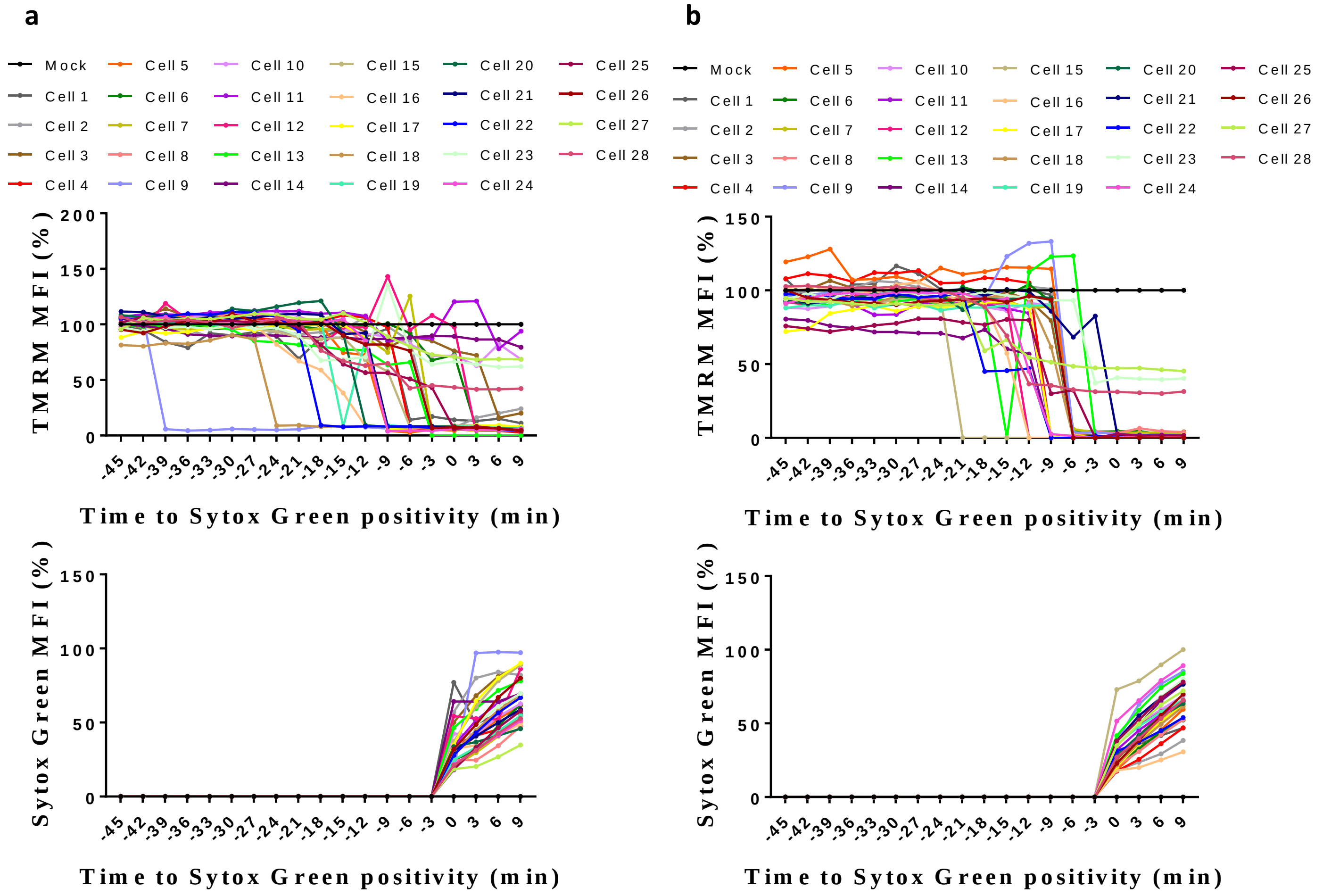
a
b
Mock
Cell 1
Cell 2
Cell 3
Cell 4
Cell 5
Cell 6
Cell 7
Cell 8
Cell 9
Cell 10
Cell 11
Cell 12
Cell 13
Cell 14
Cell 15
Cell 16
Cell 17
Cell 18
Cell 19
Cell 20
Cell 21
Cell 22
Cell 23
Cell 24
Cell 25
Cell 26
Cell 27
Cell 28
TMRM MFI (%)
Sytox Green MFI (%)
Time to Sytox Green positivity (min)

Suppl Fig 3

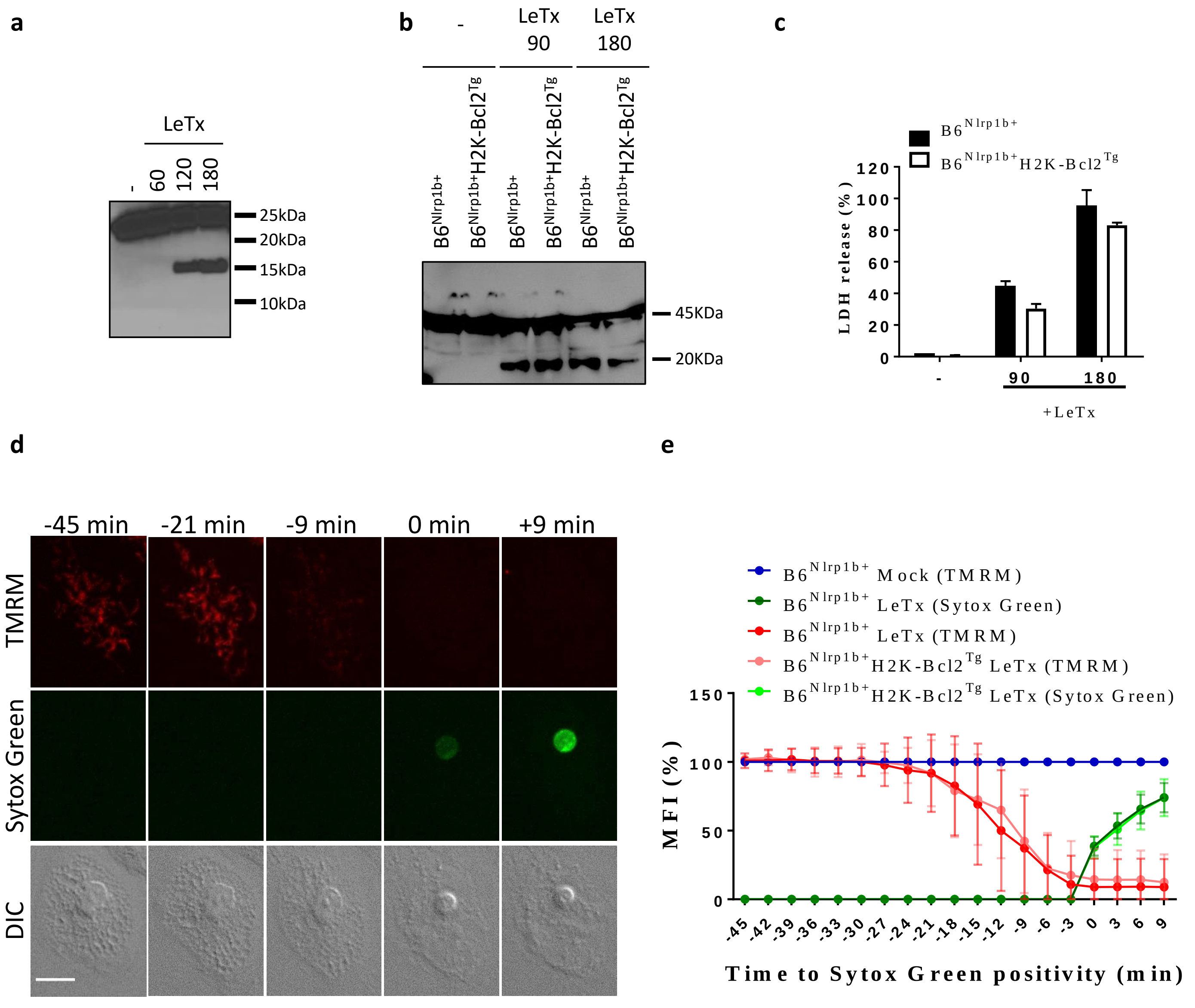
a
LeTx
-
60
120
180
25kDa
20kDa
15kDa
10kDa
b
-
LeTx
90
LeTx
180
B6Nlrp1b+
B6Nlrp1b+H2K-Bcl2Tg
45KDa
20KDa
c
B6Nlrp1b+
B6Nlrp1b+H2K-Bcl2Tg
LDH release (%)
0
20
40
60
80
100
120
-
90
180
+LeTx
d
-45 min
-21 min
-9 min
0 min
+9 min
TMRM
Sytox Green
DIC
e
B6Nlrp1b+ Mock (TMRM)
B6Nlrp1b+ LeTx (Sytox Green)
B6Nlrp1b+ LeTx (TMRM)
B6Nlrp1b+H2K-Bcl2Tg LeTx (TMRM)
B6Nlrp1b+H2K-Bcl2Tg LeTx (Sytox Green)
MFI (%)
0
50
100
150
-45 -42 -39 -36 -33 -30 -27 -24 -21 -18 -15 -12 -9 -6 -3 0 3 6 9
Time to Sytox Green positivity (min)

Suppl Fig 4

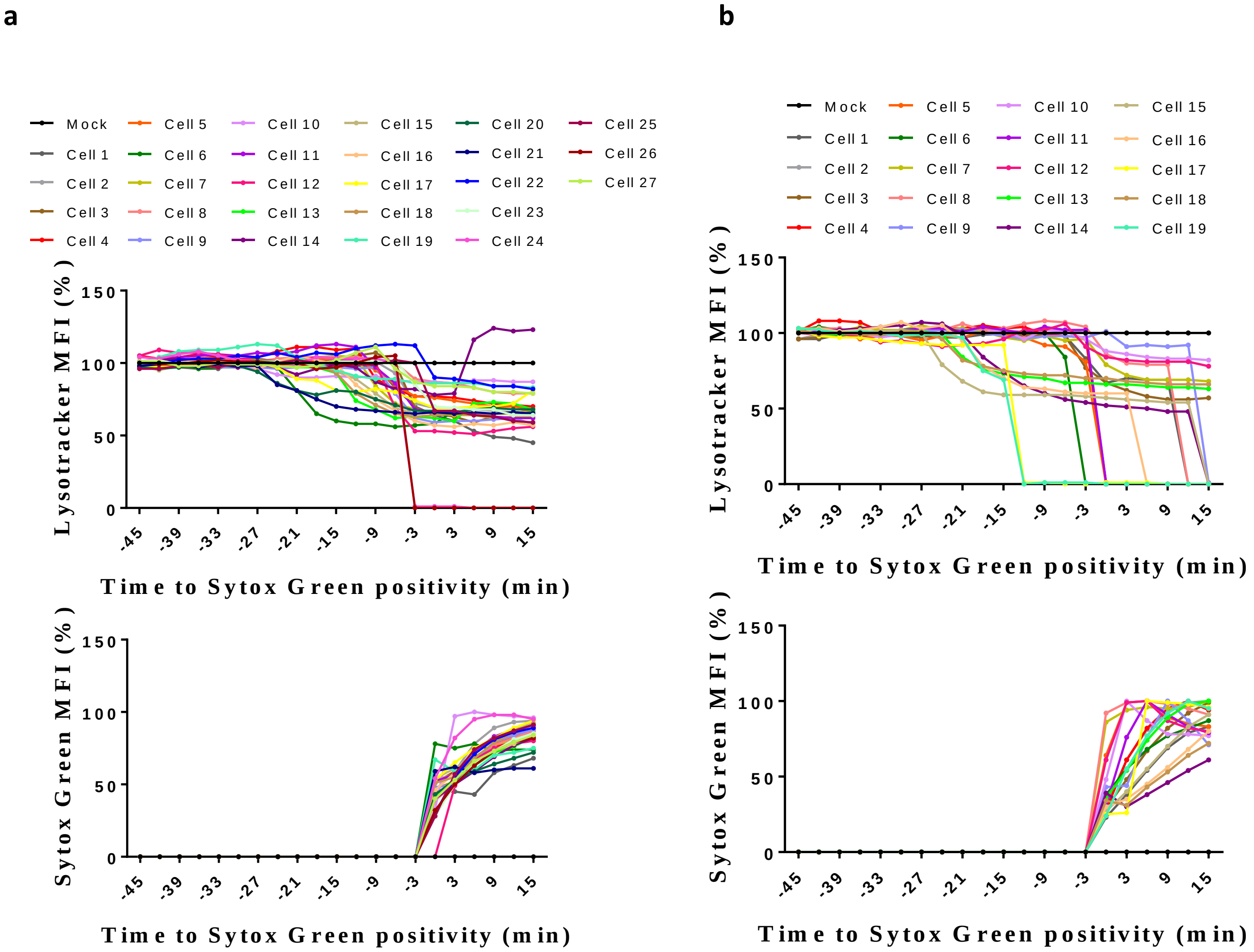
a
b
Mock
Cell 1
Cell 2
Cell 3
Cell 4
Cell 5
Cell 6
Cell 7
Cell 8
Cell 9
Cell 10
Cell 11
Cell 12
Cell 13
Cell 14
Cell 15
Cell 16
Cell 17
Cell 18
Cell 19
Cell 20
Cell 21
Cell 22
Cell 23
Cell 24
Cell 25
Cell 26
Cell 27
Lysotracker MFI (%)
Sytox Green MFI (%)
Time to Sytox Green positivity (min)
150
100
50
0
-45
-39
-33
-27
-21
-15
-9
-3
3
9
15

Suppl Fig 5

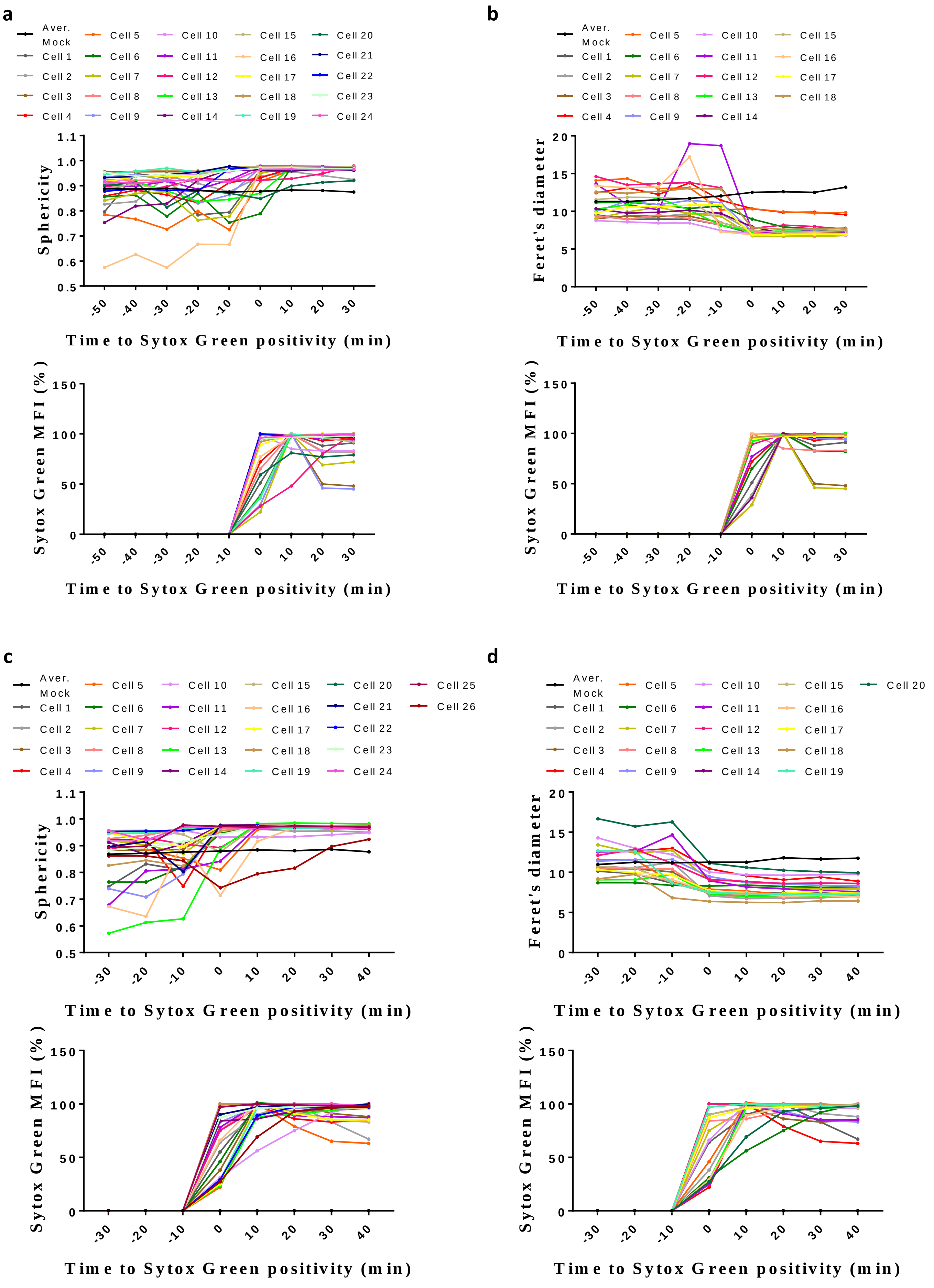
a
b
c
d
Aver. Mock
Cell 1
Cell 2
Cell 3
Cell 4
Cell 5
Cell 6
Cell 7
Cell 8
Cell 9
Cell 10
Cell 11
Cell 12
Cell 13
Cell 14
Cell 15
Cell 16
Cell 17
Cell 18
Cell 19
Cell 20
Cell 21
Cell 22
Cell 23
Cell 24
Cell 25
Cell 26
Sphericity
Feret's diameter
Sytox Green MFI (%)
Time to Sytox Green positivity (min)

Suppl Fig 6

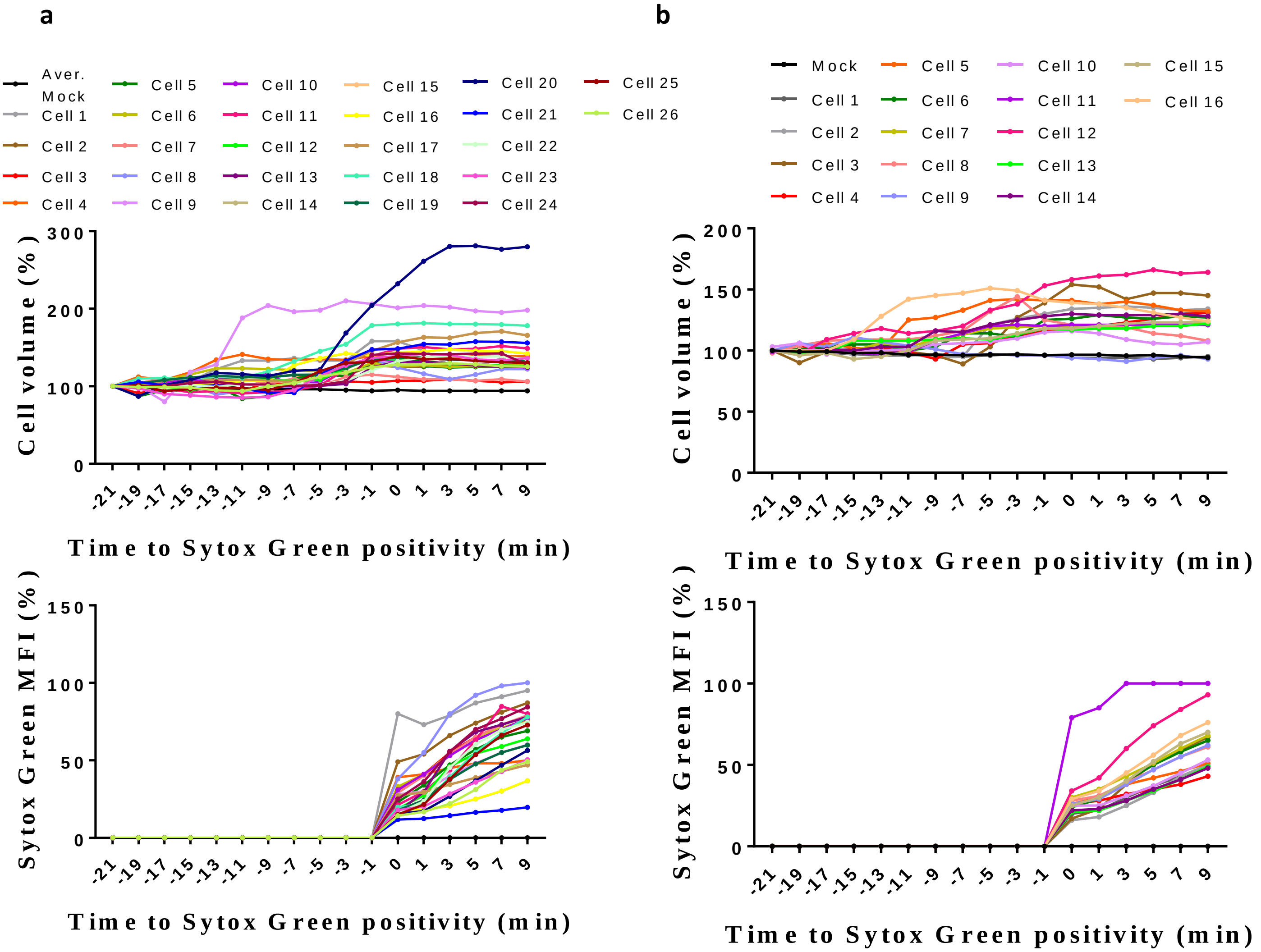

a
Aver. Mock
Cell 1
Cell 2
Cell 3
Cell 4
Cell 5
Cell 6
Cell 7
Cell 8
Cell 9
Cell 10
Cell 11
Cell 12
Cell 13
Cell 14
Cell 15
Cell 16
Cell 17
Cell 18
Cell 19
Cell 20
Cell 21
Cell 22
Cell 23
Cell 24
Cell 25
Cell 26
Cell volume (%)
Sytox Green MFI (%)
Time to Sytox Green positivity (min)
b
Mock
Cell volume (%)
Sytox Green MFI (%)
Time to Sytox Green positivity (min)

Suppl Fig 7

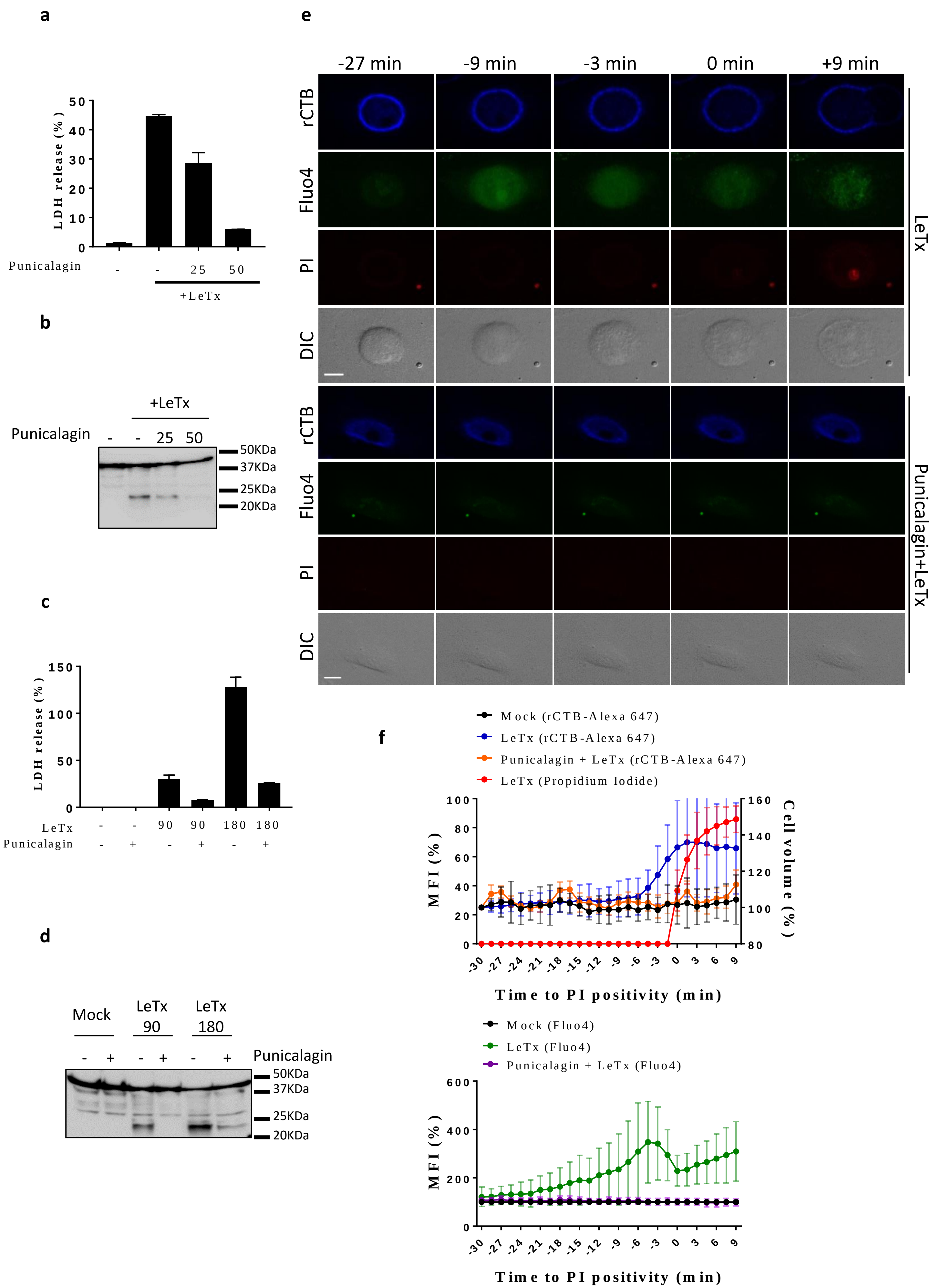
a
LDH release (%)
50
40
30
20
10
0
Punicalagin
-
-
25
50
+LeTx
b
+LeTx
Punicalagin
-
-
25
50
50KDa
37KDa
25KDa
20KDa
c
LDH release (%)
150
100
50
0
LeTx
-
-
90
90
180
180
Punicalagin
-
+
-
+
-
+
d
Mock
LeTx 90
LeTx 180
-
+
-
+
-
+
Punicalagin
50KDa
37KDa
25KDa
20KDa
e
-27 min
-9 min
-3 min
0 min
+9 min
rCTB
Fluo4
PI
DIC
LeTx
Punicalagin+LeTx
f
Mock (rCTB-Alexa 647)
LeTx (rCTB-Alexa 647)
Punicalagin + LeTx (rCTB-Alexa 647)
LeTx (Propidium Iodide)
MFI (%)
100
80
60
40
20
0
Cell volume (%)
160
140
120
100
80
-30
-27
-24
-21
-18
-15
-12
-9
-6
-3
0
3
6
9
Time to PI positivity (min)
Mock (Fluo4)
LeTx (Fluo4)
Punicalagin + LeTx (Fluo4)
MFI (%)
600
400
200
0
-30
-27
-24
-21
-18
-15
-12
-9
-6
-3
0
3
6
9
Time to PI positivity (min)

Suppl Fig 8

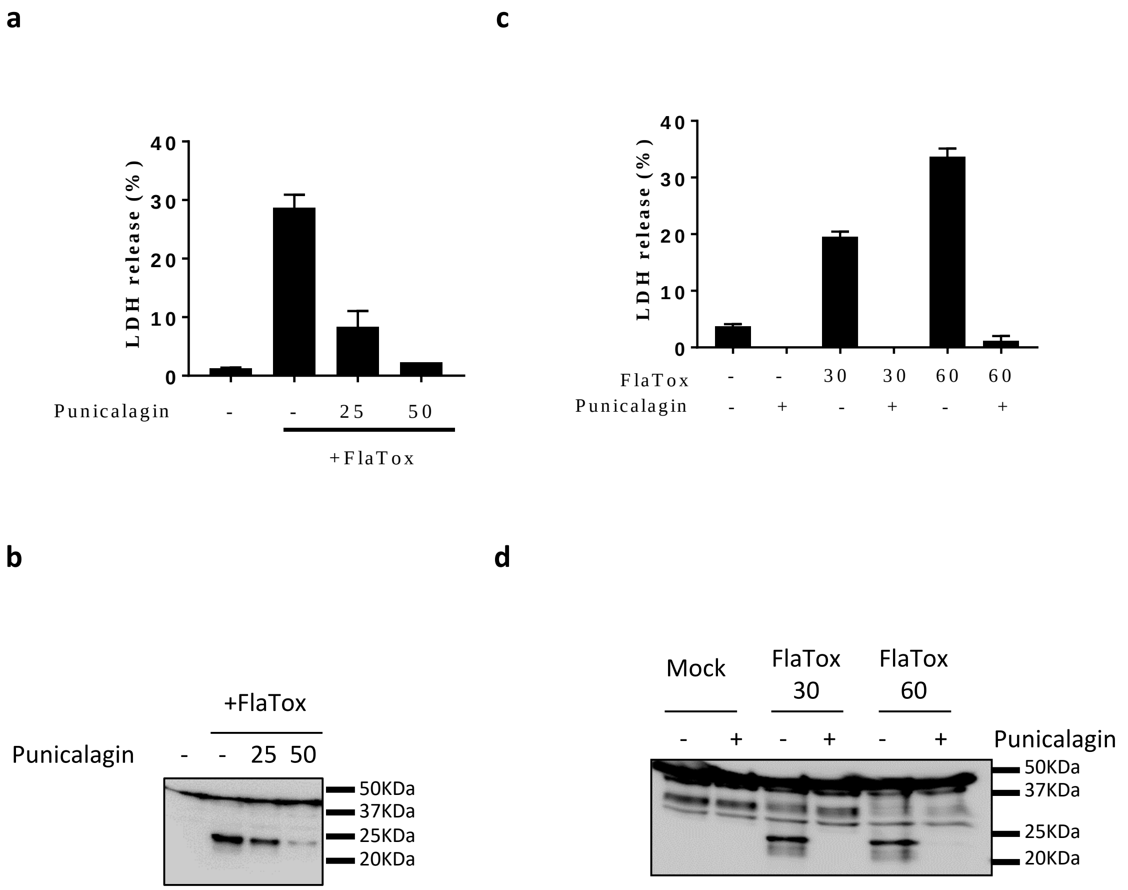
a
LDH release (%)
40
30
20
10
0
Punicalagin
-
-
25
50
+FlaTox
b
+FlaTox
Punicalagin
-
-
25
50
50KDa
37KDa
25KDa
20KDa
c
LDH release (%)
40
30
20
10
0
FlaTox
-
-
30
30
60
60
Punicalagin
-
+
-
+
-
+
d
Mock
FlaTox 30
FlaTox 60
-
+
-
+
-
+
Punicalagin
50KDa
37KDa
25KDa
20KDa

Suppl Fig 9

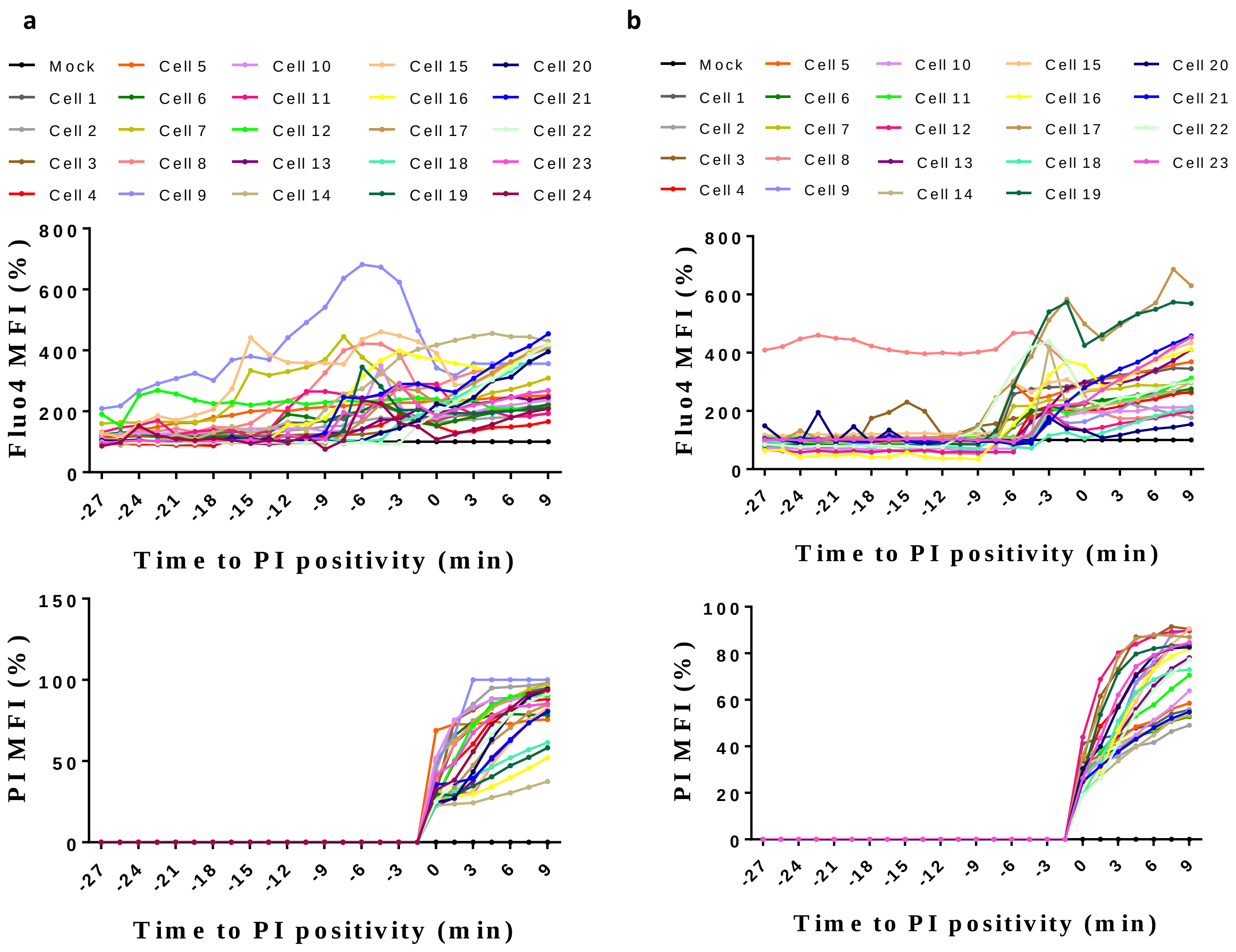
a
b
Mock
Cell 1
Cell 2
Cell 3
Cell 4
Cell 5
Cell 6
Cell 7
Cell 8
Cell 9
Cell 10
Cell 11
Cell 12
Cell 13
Cell 14
Cell 15
Cell 16
Cell 17
Cell 18
Cell 19
Cell 20
Cell 21
Cell 22
Cell 23
Cell 24
Fluo4 MFI (%)
PI MFI (%)
Time to PI positivity (min)

Suppl Fig 10

a

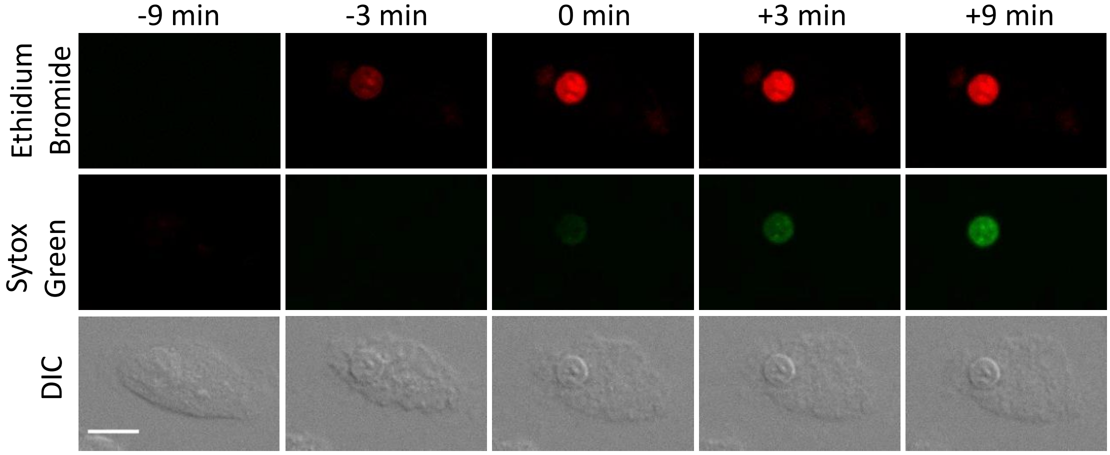

b

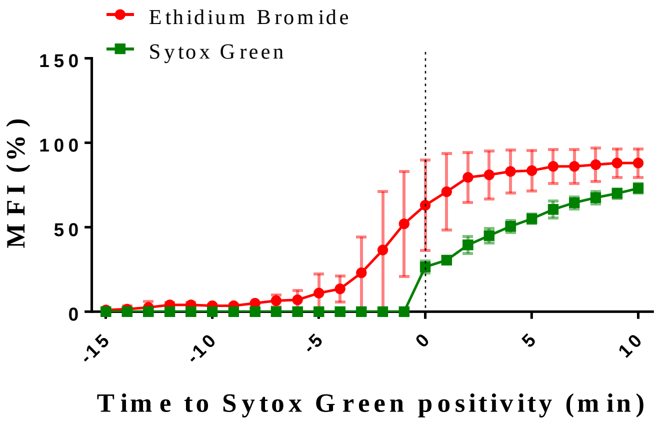

Suppl Fig 11

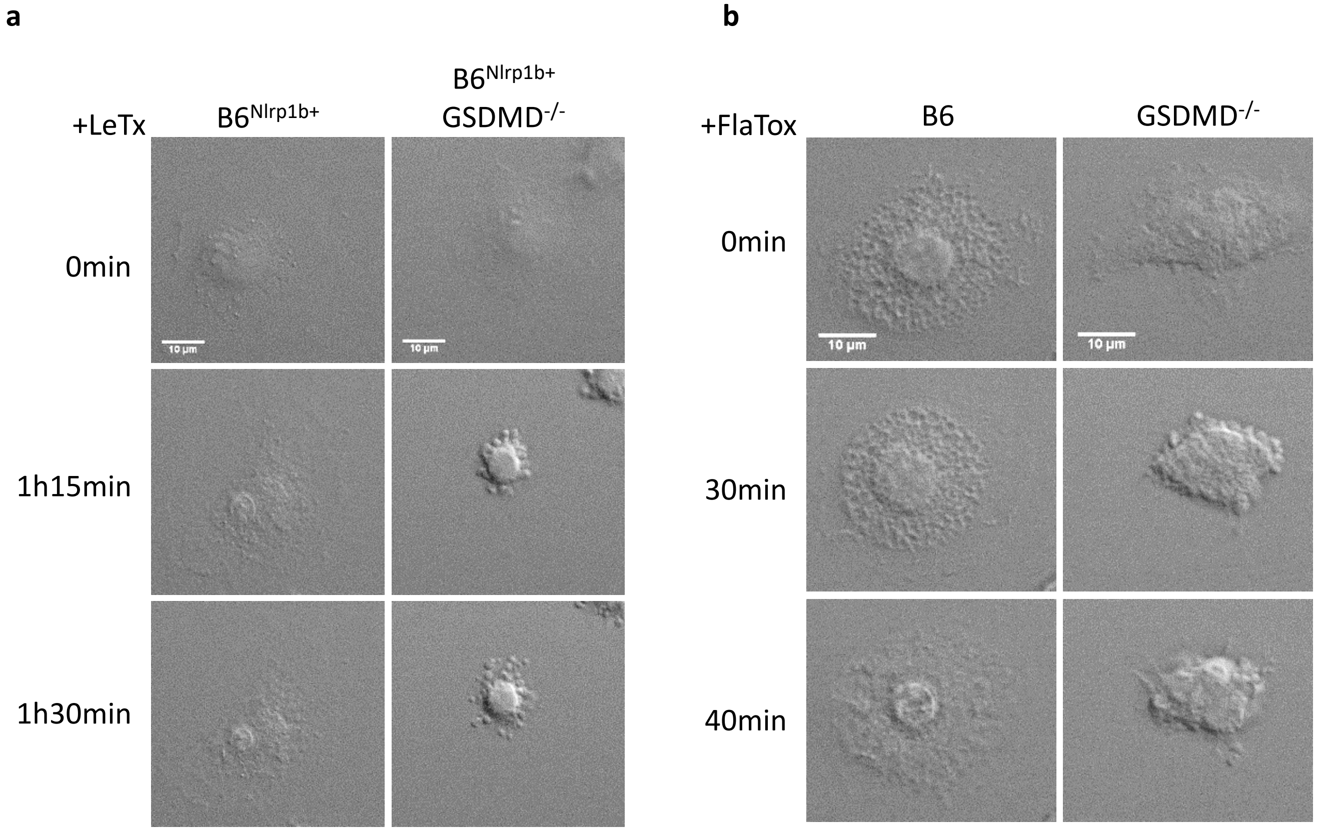
a
+LeTx
B6Nlrp1b+
B6Nlrp1b+
GSDMD-/-
0min
1h15min
1h30min
10 μm
10 μm
b
+FlaTox
B6
GSDMD-/-
0min
30min
40min
10 μm
10 μm

Suppl Fig 12

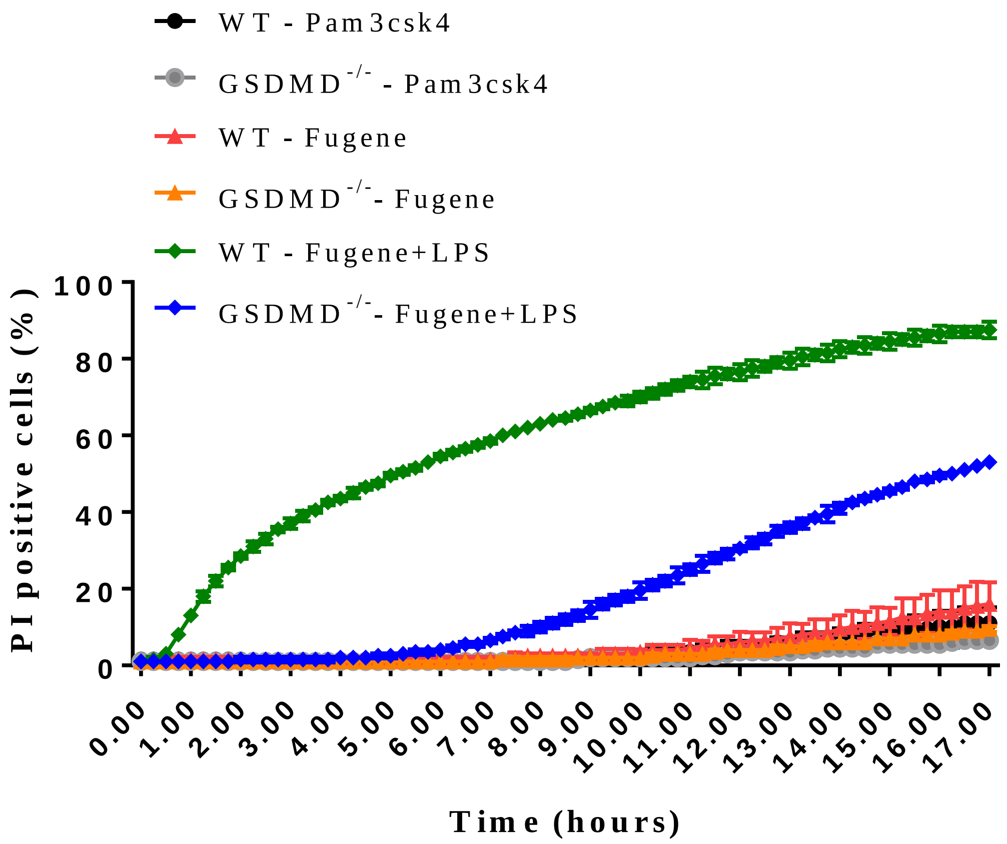

WT - Pam3csk4
GSDMD-/- - Pam3csk4
WT - Fugene
GSDMD-/- - Fugene
WT - Fugene+LPS
GSDMD-/- - Fugene+LPS
PI positive cells (%)
100
80
60
40
20
0
0.00
1.00
2.00
3.00
4.00
5.00
6.00
7.00
8.00
9.00
10.00
11.00
12.00
13.00
14.00
15.00
16.00
17.00
Time (hours)

Suppl Fig 13

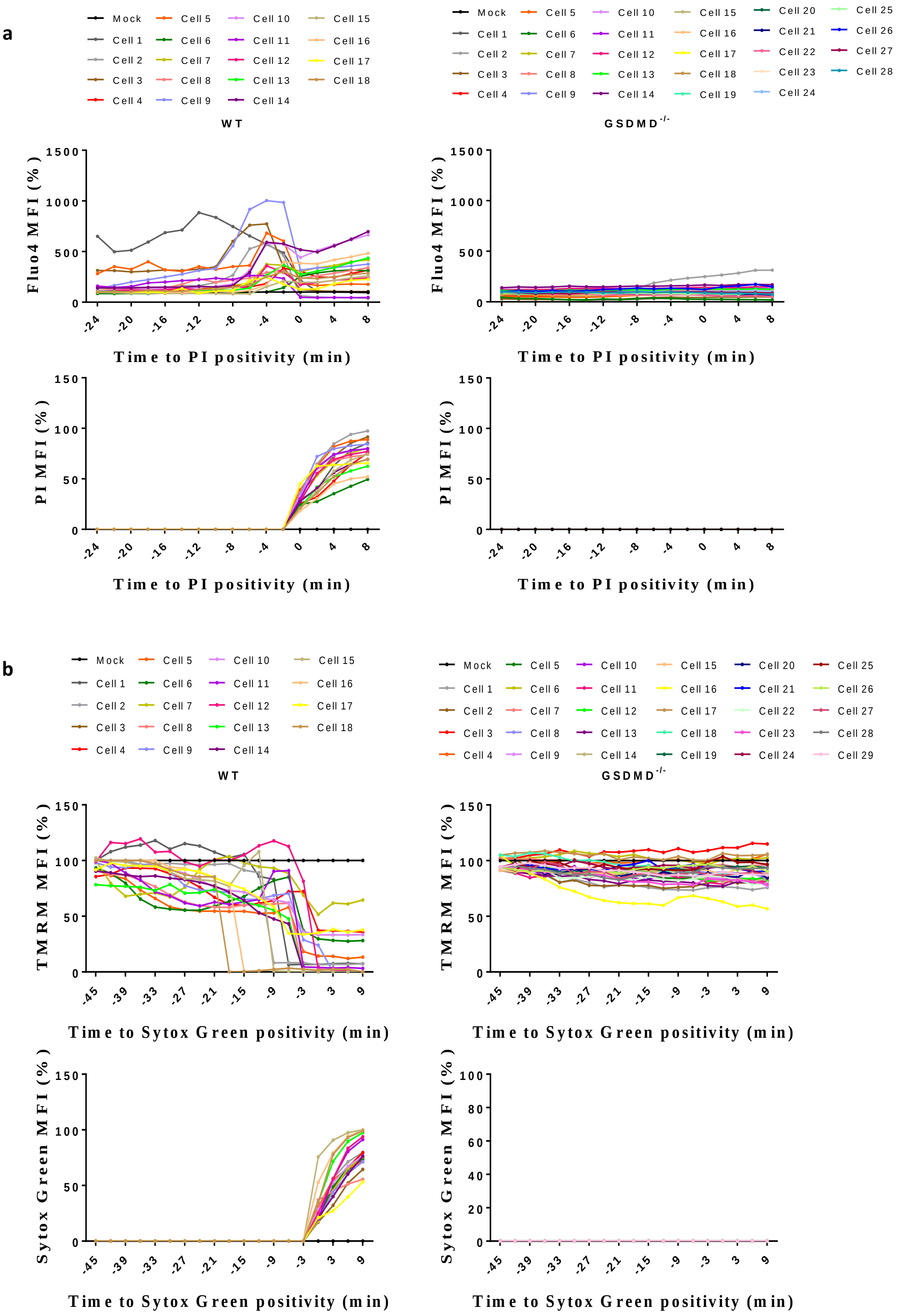
a
Mock
Cell 1
Cell 2
Cell 3
Cell 4
Cell 5
Cell 6
Cell 7
Cell 8
Cell 9
Cell 10
Cell 11
Cell 12
Cell 13
Cell 14
Cell 15
Cell 16
Cell 17
Cell 18
WT
Cell 19
Cell 20
Cell 21
Cell 22
Cell 23
Cell 24
Cell 25
Cell 26
Cell 27
Cell 28
GSDMD-/-
Fluo4 MFI (%)
Time to PI positivity (min)
PI MFI (%)
b
Cell 29
TMRM MFI (%)
Time to Sytox Green positivity (min)
Sytox Green MFI (%)

Suppl Fig 14
