# Supplementary material for: Single-cell analysis of pyroptosis dynamics reveals conserved GSDMD-mediated subcellular events that precede plasma membrane rupture
Source: Cell Death Differ. 2018 Apr 17;26(1):146–61. doi: 10.1038/s41418-018-0106-7 (PMC6294780; doi:10.1038/s41418-018-0106-7)
Supplement: Supplementary file 2 — Supplementary Data [file 41418_2018_106_MOESM2_ESM.docx]

**Supplementary Data for:**

***Single-cell analysis of pyroptosis dynamics reveals conserved GSDMD-mediated subcellular events that precede plasma membrane rupture***

Nathalia M. de Vasconcelos^1,2^, Nina Van Opdenbosch^1,2^, Hanne Van Gorp^1,2^, Eef Parthoens^2,3^, Mohamed Lamkanfi^1,2^

*^1^ Department of Internal Medicine, Ghent University, Ghent, B-9000, Belgium*

*^2^ VIB-UGhent Center for Inflammation Research,* *VIB, Ghent, B-9000, Belgium*

*^3^ VIB Bioimaging Core, VIB, Ghent, B-9000, Belgium*

**Supplementary Figure Legends**

**Supplementary Movie Legends**

**Supplementary Figures 1-14**

**Supplementary Movies 1-19**

**Supplementary Figure Legends**

**Supplemental Figure 1. PS exposure happens during pyroptosis**. **a-b,** BMDMs were stimulated with LeTx (**a**) or FlaTox (**b**) and imaged in culture media containing Annexin-V-FITC and PI. Confocal images were acquired every 3 minutes. Graphs show the percentage of mean fluorescence intensity (MFI) of single cells (LeTx n=18; FlaTox n=21), calculated as described in *Online* *Methods*, of Annexin-V (upper panel) or PI (lower panel) signals. In all panels time point zero indicates the first detection of PI. Relates to Figure 2b, d.

**Supplemental Figure 2. Mitochondrial morphology of mock-treated BMDMs.** BMDMs preloaded with Mitotracker Red CMXRos were imaged in culture media containing Sytox Green (n=50). A single plane of a representative cell is shown. Scale bars, 10 µm.

**Supplemental Figure 3. Mitochondria are damaged during pyroptosis.** **a-b,** BMDMs were preloaded with TMRM and stimulated with either LeTx (**a**) or FlaTox (**b**) and imaged in culture media containing Sytox Green. Confocal images were acquired every 3 minutes. Graphs show the percentage of mean fluorescence intensity (MFI) of single cells (LeTx, n=28; FlaTox n=28), calculated as described in *Online* *Methods*, of TMRM (upper panel) or Sytox Green (lower panel) signals. “Mock” lines represent the average of the values obtained in unstimulated cells, imaged in parallel. In all panels time point zero indicates the first detection of Sytox Green. Relates to Figure 3c, e.

**Supplemental Figure 4. Bax/Bak pores are dispensable for pyroptosis-associated mitochondrial damage. a**, Protein lysates of BMDMs stimulated with LeTx for 60, 120 or 180 minutes were analyzed by Western blotting for BID. **a-c**, Protein lysate of B6 ^Nlrp1b+^ (WT) and B6 ^Nlrp1b+/H2kBcl2+^ (H2K-Bcl2^Tg^) BMDMs that have been stimulated with LeTx for 90 or 180 minutes were assayed by Western blotting for caspase-1 maturation (**b**), and their culture supernatants were assayed for LDH activity (**c**). **d, e**, B6 ^Nlrp1b+^ (WT) and B6 ^Nlrp1b+/H2kBcl2+^ (H2K-Bcl2^Tg^) BMDMs were loaded with TMRM and stimulated with LeTx in culture media containing Sytox Green. Confocal images were acquired every 3 minutes. Graphs show the percentage of mean fluorescence intensity (MFI) calculated as described in *Online Methods*, and values represent the mean ± SD of individual cells imaged in three independent experiments (WT n=26; H2K-Bcl2^Tg^ n=21). Fluorescent micrographs show the maximum intensity projection of a representative cell. In panels **d, e** time point zero indicates the first detection of Sytox Green. All scale bars, 10 µm.

**Supplemental Figure 5. Lysosomes decay prior to pyroptotic cell lysis. a-b,** BMDMs preloaded with Lysotracker and stimulated with LeTx (**a**) or FlaTox (**b**) were imaged throughout cell death in culture media containing Sytox Green. Confocal images were taken every 3 minutes. Graphs show the percentage of mean fluorescence intensity (MFI) of single cells (LeTx, n=27; FlaTox n=19), calculated as described in *Online* *Methods*, of Lysotracker (upper panel) or Sytox Green (lower panel) signals. “Mock” lines represent the average of the values obtained in unstimulated cells, imaged in parallel. In all panels time point zero indicates the first detection of Sytox Green. Relates to Figure 4b, d.

**Supplemental Figure 6.** **Nuclei round up and condense during pyroptosis. a-d,** BMDMs were preloaded with Hoechst dye and stimulated with LeTx (**a, b**) or FlaTox (**c, d**) before imaging in culture media containing Sytox Green. Confocal images were acquired every 10 minutes. Graphs show values for nuclear sphericity (**a, c**, upper panels) or Feret’s diameter (**b, d**, upper panels) based on Hoechst staining or the percentage of mean fluorescence intensity (MFI) of Sytox Green signal (lower panels) of single cells (LeTx: Sphericity n=24, Feret’s diameter n= 18; FlaTox: Sphericity n=26, Feret’s diameter n=20), calculated as described in *Online* *Methods*. “Mock” lines represent the average of the values obtained in unstimulated cells, imaged in parallel. In all panels time point zero indicates the first detection of Sytox Green. Relates to Figure 5b, c, d, e.

**Supplemental Figure 7. Cell swelling precedes pyroptotic cell rupture.** **a,b,** BMDMs stained with Cholera Toxin subunit B-Alexa 594 (rCTB) were stimulated with LeTx (**a**) or FlaTox (**b**) and imaged in culture media containing Sytox Green. Confocal images were acquired every 1.5 minutes. Graphs show the percentage of cell volume quantifications based on rCTB-Alexa 594 staining (upper panel) or the mean fluorescence intensity (MFI) of Sytox Green signal (lower panels) of single cells (LeTx, n=26; FlaTox n=16), calculated as described in *Online* *Methods*. “Mock” lines represent the average of the values obtained in unstimulated cells, imaged in parallel. In all panels time point zero indicates the first detection of Sytox Green. Relates to Figure 6b, d.

**Supplemental Figure 8. Punicalagin inhibits LeTx-induced pyroptosis upstream of caspase-1 activation.** **a, b,** BMDMs were pretreated with Punicalagin at the indicated concentrations (µM) and stimulated with LeTx for 90 min. Culture supernatants were assayed for LDH (**a**), and protein lysates for caspase-1 by Western blotting (**b**). (**c, d**) BMDMs were pretreated with Punicalagin (50 µM) and stimulated with LeTx for 90 or 180 minutes. Culture supernatants were assayed for LDH (**c**), and protein lysates for caspase-1 by Western blotting (**d**). (**e, f**) BMDMs that had been preloaded with the Ca^2+^ indicator Fluo4 and stained with CTB-Alexa 647 (rCTB) were incubated with Punicalagin (50 µM) or vehicle control before cells were stimulated with LeTx and imaged in culture media containing PI. Confocal images were acquired every 1.5 minutes. Graphs show the percentage of mean fluorescence intensity (MFI) of PI (upper panel, left axis) and Fluo4 (lower panel) and cell volume quantifications based on rCTB-Alexa 647 staining (upper panel, right axis), all calculated as described in *Online Methods*. Values represent the mean ± SD of two independent experiments (LeTx n=11; Punicalagin+LeTx n=9). Fluorescent micrographs show the maximum intensity projection (PI and Fluo4) or the single plane (rCTB) of a representative cell. All scale bars, 10 µm.

**Supplemental Figure 9. Punicalagin inhibits FlaTox-induced caspase-1 activation.** **a, b**, BMDMs were pretreated with Punicalagin at the indicated concentrations and stimulated with FlaTox for 30 min. Culture supernatants were assayed for LDH (**a**), and protein lysates for caspase-1 by Western blotting (**b**). BMDMs were pretreated with Punicalagin (50 µM) and stimulated with FlaTox for 30 or 60 minutes. Culture supernatants were assayed for LDH (**c**), and protein lysates for caspase-1 by Western blotting (**d**). Data are representative of two independent experiments.

**Supplemental Figure 10. Ca^2+^ influx occurs prior to total membrane permeabilization in pyroptosis**.  **a-d,** BMDMs preloaded with the cell-permeant Ca^2+^ indicator Fluo4 were imaged after stimulation with LeTx (**a**) or FlaTox (**b**) in culture media containing PI. Confocal images were acquired every 1.5 minutes. Graphs show the percentage of mean fluorescence intensity (MFI) of single cells (LeTx, n=24; FlaTox n=23), calculated as described in *Online* *Methods*, of Fluo4 (upper panel) or PI (lower panel) signals. “Mock” lines represent the average of the values obtained in unstimulated cells, imaged in parallel. In all panels time point zero indicates the first detection of PI. Relates to Figure 7b, d.

**Supplemental Figure 11. Pyroptotic cells have differential permeability to Ethidium Bromide and Sytox Green.** **a, b,** BMDMs were stimulated with LeTx and imaged in culture media containing Ethidium Bromide and Sytox Green. Confocal images were acquired every minute. Graph shows the percentage of mean fluorescence intensity (MFI) calculated as described in *Online* *Methods*, and values represent the mean ± SD of individual cells imaged in three independent experiments (n=50). Fluorescent micrographs show the maximum intensity projection of a representative cell. In all panels time point zero indicates the first detection of Sytox Green. All scale bars, 10 µm.

**Supplemental Figure 12.** **Gasdermin D-deficient macrophages die by apoptosis with same kinetics as WT undergo pyroptosis in response to canonical inflammasome stimuli.** **a,b,** GSDMD-deficient and –sufficient B6 and B6^Nlrp1b+^ BMDMs were stimulated with LeTx (**a**) or FlaTox (**b**). Images show the bright field of representative cells (LeTx n=20; FlaTox n=18). All scale bars, 10 µm.

**Supplemental Figure 13. Gasdermin D-deficient macrophages demonstrate a delayed membrane permeabilization after non-canonical inflammasome activation.** Pam3-csk4-primed GSDMD-deficient and –sufficient B6 BMDMs were transfected with LPS (Fugene+LPS), treated with Fugene alone or kept without treatment and imaged on an INCUCYTE in media containing PI. The number of positive cells was quantified relative to a Triton-x100-treated well (considered 100%) of each genotype. Values represent mean ± SD of technical duplicates.

**Supplemental Figure 14. Lack of GSDMD recues the Ca^2+^ influx and mitochondrial decay after activation of the non-canonical inflammasome****. a,b,** Pam3-csk4-primmed BMDMs of either WT (**a**) or GSDMD^-/-^ (**b**) mice were preloaded with the cell-permeant Ca^2+^ indicator Fluo4 and imaged after transfection with LPS (Fugene+LPS), treated Fugene alone or “mock” treated in culture media containing PI. Confocal images were acquired every 2 minutes. **c,d,** BMDMs of either WT (**c**) or GSDMD^-/-^ (**d**) mice were preloaded with TMRM and imaged after transfection with LPS (Fugene+LPS), treated Fugene alone or “mock” treated in culture media containing Sytox Green. Confocal images were acquired every 3 minutes. Graphs show the percentage of mean fluorescence intensity (MFI) of single cells (Fluo4: WT n=18, GSDMD^-/-^ n=28; TMRM: WT n=18, GSDMD^-/-^ n=29), calculated as described in *Online* *Methods*, of Fluo4 and TMRM (upper panels) or PI and Sytox Green (lower panels) signals. “Mock” lines represent the average of the values obtained in unstimulated cells, imaged in parallel. In all panels time point zero indicates the first detection of PI. Relates to Figure 8d, f.

**Supplementary Movie Legends**

**Supplemental Movie 1.** Necroptotic cells detach and round up before becoming Sytox Green positive. BMDMs were stimulated with TNF+BV6+zVAD-fmk and imaged in culture media containing Sytox Green. Confocal images were acquired every three minutes. Fluorescent micrographs show the maximum intensity projection (Sytox Green) of a representative cell from 30 analysed cells in four independent experiments. Time point zero indicates the start of imaging. Relates to Figure 1a. Scale bar, 10 µm.

**Supplemental Movie 2.** Pyroptotic cells remain attached during membrane permeabilization. Cholera Toxin subunit B-Alexa 647 (rCTB) stained BMDMs were stimulated with LeTx and imaged in culture media containing Sytox Green. Confocal images were acquired every three minutes. Fluorescent micrographs show the maximum intensity projection (Sytox Green) or the single plane (rCTB) of a representative cell from 30 analysed cells in three independent experiments. Time point zero indicates the start of imaging. Relates to Figure 1b. Scale bar, 10 µm.

**Supplemental Movie 3.** Phosphatidylserine is exposed during LeTx-induced pyroptosis. BMDMs were stimulated with LeTx and imaged in culture media containing Annexin-V-FITC and PI. Confocal images were acquired every 3 minutes. Fluorescent micrographs show the maximum intensity projection of a representative cell from 18 analysed cells in three independent experiments. Time point zero indicates the start of imaging. Relates to Figure 2a. Scale bar, 10 µm.

**Supplemental Movie 4.** Mock-treated BMDMs are not stained with Annexin-V nor PI during imaging. BMDMs mock treated were imaged in culture media containing Annexin-V-FITC and PI. Confocal images were acquired every 3 minutes. Fluorescent micrographs show the maximum intensity projection of a representative cell from 25 analysed cells in three independent experiments. Time point zero indicates the start of imaging. Relates to Figure 2. Scale bar, 10 µm.

**Supplemental Movie 5.** FlaTox-induced pyroptosis also terminates with phosphatidylserine exposure. BMDMs were stimulated with FlaTox and imaged in culture media containing Annexin-V-FITC and PI. Confocal images were acquired every 3 minutes. Fluorescent micrographs show the maximum intensity projection of a representative cell from 21 analysed cells in three independent experiments. Time point zero indicates the start of imaging. Relates to Figure 2c. Scale bar, 10 µm.

**Supplemental Movie 6.** Changes in mitochondrial morphology accompany LeTx-induced pyroptosis. BMDMs preloaded with Mitotracker Red CMXRos were either mock (upper panel) or LeTx-stimulated (lower panel) and imaged in culture media containing Sytox Green. Confocal images were acquired every 3 minutes. Fluorescent micrographs show the maximum intensity projection (Sytox Green) or the single plane (Mitotracker) of a representative cell from 50 analysed cells in three independent experiments. Time point zero indicates the start of imaging. Relates to Figure 3a. Scale bar, 10 µm.

**Supplemental Movie 7.** Mitochondria decay during pyroptosis in LeTx-stimulated cells. BMDMs preloaded with TMRM were either mock (upper panel) or LeTx-treated (lower panel) and imaged in culture media containing Sytox Green. Confocal images were acquired every 3 minutes. Fluorescent micrographs show the maximum intensity projection of a representative cell from 28 analysed cells in five independent experiments. Time point zero indicates the start of imaging. Relates to Figure 3b. Scale bar, 10 µm.

**Supplemental Movie 8.** Pyroptosis triggered with FlaTox also occurs with mitochondrial decay. BMDMs preloaded with TMRM were either mock (upper panel) or FlaTox-treated (lower panel) and imaged in culture media containing Sytox Green. Confocal images were acquired every 3 minutes. Fluorescent micrographs show the maximum intensity projection of a representative cell out from 28 analysed cells in five independent experiments. Time point zero indicates the start of imaging. Relates to Figure 3d. Scale bar, 10 µm.

**Supplemental Movie 9.** Lysosomes decay during pyroptosis triggered by LeTx. BMDMs preloaded with Lysotracker were either mock (upper panel) or LeTx-treated (lower panel) and imaged in culture media containing Sytox Green. Confocal images were taken every 3 minutes. Fluorescent micrographs show the maximum intensity projection of a representative cell from 27 analysed cells in three independent experiments. Time point zero indicates the start of imaging. Relates to Figure 4a. Scale bar, 10 µm.

**Supplemental Movie 10.** Lysosomal decay accompanies pyroptosis triggered by FlaTox. BMDMs preloaded with Lysotracker were either mock (upper panel) or FlaTox-treated (lower panel) and imaged in culture media containing Sytox Green. Confocal images were taken every 3 minutes. Fluorescent micrographs show the maximum intensity projection of a representative cell from 19 analysed cells in three independent experiments. Time point zero indicates the start of imaging. Relates to Figure 4c. Scale bar, 10 µm.

**Supplemental Movie 11.** Nuclei undergo morphology alterations during LeTx-induced pyroptosis. BMDMs preloaded with Hoechst were either mock (upper panel) or LeTx-treated (lower panel) before being imaged in culture media containing Sytox Green. Confocal images were acquired every 10 minutes. Fluorescent micrographs show the maximum intensity projection of a representative cell from 24 analysed cells in three independent experiments. Time point zero indicates the start of imaging. Relates to Figure 5a. Scale bar, 10 µm.

**Supplemental Movie 12.** Nuclear alterations also follow FlaTox-triggered pyroptosis. BMDMs preloaded with Hoechst were either mock (upper panel) or FlaTox-treated (lower panel) before being imaged in culture media containing Sytox Green. Confocal images were acquired every 10 minutes. Fluorescent micrographs show the maximum intensity projection of a representative cell from 26 analysed cells in three independent experiments. Time point zero indicates the start of imaging. Relates to Figure 5c. Scale bar, 10 µm.

**Supplemental Movie 13.** Cell volume increases gradually in LeTx-triggered pyroptosis. BMDMs stained with Cholera Toxin subunit B-Alexa 594 (rCTB) were either mock (upper panel) or LeTx-treated (lower panel) and imaged in culture media containing Sytox Green. Confocal images were acquired every 1.5 minutes. Fluorescent micrographs show the maximum intensity projection (Sytox Green) or the single plane (rCTB) of a representative cell out from 26 analysed cells in three independent experiments. Time point zero indicates the start of imaging. Relates to Figure 4a. Scale bar, 10 µm.

**Supplemental Movie 14.** Cellular swelling is also observed during FlaTox-mediated pyroptosis. BMDMs stained with Cholera Toxin subunit B-Alexa 594 (rCTB) were either mock (upper panel) or FlaTox-treated (lower panel) and imaged in culture media containing Sytox Green. Confocal images were acquired every 1.5 minutes. Fluorescent micrographs show the maximum intensity projection (Sytox Green) or the single plane (rCTB) of a representative cell from 16 analysed cells in three independent experiments. Time point zero indicates the start of imaging. Relates to Figure 4c. Scale bar, 10 µm.

**Supplemental Movie 15.** Ca^2+^ influx occurs during LeTx-induced pyroptosis and it is prior to total membrane permeabilization. BMDMs preloaded with the cell-permeant Ca^2+^ indicator Fluo4 were either mock (upper panel) or LeTx-treated (lower panel) and imaged in culture media containing PI. Confocal images were acquired every 1.5 minutes. Fluorescent micrographs show the maximum intensity projection of a representative cell from 24 analysed cells in four independent experiments. Time point zero indicates the start of imaging. Relates to Figure 6a. Scale bar, 10 µm.

**Supplemental Movie 16.** FlaTox-induced pyroptosis also happens with Ca^2+^ influx prior to total membrane rupture. BMDMs preloaded with the cell-permeant Ca^2+^ indicator Fluo4 were either mock (upper panel) or FlaTox-treated (lower panel) and imaged in culture media containing PI. Confocal images were acquired every 1.5 minutes. Fluorescent micrographs show the maximum intensity projection of a representative cell from 23 analysed cells in four independent experiments. Time point zero indicates the start of imaging. Relates to Figure 6c. Scale bar, 10 µm.

**Supplemental Movie 17.** Differential incorporation of Ethidium Bromide and Sytox Green in pyroptotic cells. BMDMs were stimulated with LeTx and imaged in culture media containing Ethidium Bromide and Sytox Green. Confocal images were acquired every minute. Fluorescent micrographs show the maximum intensity projection of a representative cell from 50 analysed cells in three independent experiments. Time point zero indicates the start of imaging. Relates to Supplemental Figure 5a. Scale bar, 10 µm.

**Supplemental Movie 18.** Ca^2+^ influx also precedes total membrane permeabilization after non-canonical inflammasome activation and it is rescued by lack of GSDMD. Pam3-csk4-primmed BMDMs of either WT (upper panel) or GSDMD^-/-^ (lower panel) mice were preloaded with the cell-permeant Ca^2+^ indicator Fluo4 and transfected with LPS for 1h30 before imaging in culture media containing PI. Confocal images were acquired every 2 minutes. Fluorescent micrographs show the maximum intensity projection of a representative cell from 18 (WT) or 28 (GSDMD^-/-^) analysed cells in three independent experiments. Time point zero indicates the start of imaging. Relates to Figure 8a, b. Scale bar, 10 µm.

**Supplemental Movie 19.** Lack of GSDMD rescues mitochondrial decay during pyroptosis in LPS-transfected cells. Pam3-csk4-primmed BMDMs of either WT (upper panel) or GSDMD^-/-^ (lower panel) mice were preloaded with TMRM and imaged in culture media containing Sytox Green. Confocal images were acquired every 3 minutes. Fluorescent micrographs show the maximum intensity projection of a representative cell from 18 (WT) or 29 (GSDMD^-/-^) analysed cells in four independent experiments of 12-18 imaged cells. Time point zero indicates the start of imaging. Relates to Figure 8d, e. Scale bar, 10 µm.
